# Conjugation, Prodrug, and Co-Administration Strategies in Support of Nanotechnologies to Improve the Therapeutic Efficacy of Phytochemicals in the Central Nervous System

**DOI:** 10.3390/pharmaceutics15061578

**Published:** 2023-05-23

**Authors:** Giovanna Rassu, Milena Sorrenti, Laura Catenacci, Barbara Pavan, Luca Ferraro, Elisabetta Gavini, Maria Cristina Bonferoni, Paolo Giunchedi, Alessandro Dalpiaz

**Affiliations:** 1 Department of Medicine, Surgery and Pharmacy, University of Sassari, Via Muroni 23a, I-07100 Sassari, Italy; grassu@uniss.it (G.R.); eligav@uniss.it (E.G.); pgiunc@uniss.it (P.G.); 2 Department of Drug Sciences, University of Pavia, Viale Taramelli 12, I-27100 Pavia, Italy; milena.sorrenti@unipv.it (M.S.); laura.catenacci@unipv.it (L.C.); cbonferoni@unipv.it (M.C.B.); 3 Department of Neuroscience and Rehabilitation—Section of Physiology, University of Ferrara, Via Borsari 46, I-44121 Ferrara, Italy; pvnbbr@unife.it; 4 Department of Life Sciences and Biotechnology, University of Ferrara, Via Borsari 46, I-44121 Ferrara, Italy; frl@unife.it; 5 Department of Chemical, Pharmaceutical and Agricultural Sciences, University of Ferrara, Via Fossato di Mortara 19, I-44121 Ferrara, Italy

**Keywords:** phytochemicals, polyphenol, essential oil, neurodegeneration, glioma, conjugation, co-administration, prodrug, nanocarriers, brain-targeting

## Abstract

Phytochemicals, produced as secondary plant metabolites, have shown interesting potential therapeutic activities against neurodegenerative diseases and cancer. Unfortunately, poor bioavailability and rapid metabolic processes compromise their therapeutic use, and several strategies are currently proposed for overcoming these issues. The present review summarises strategies for enhancing the central nervous system’s phytochemical efficacy. Particular attention has been paid to the use of phytochemicals in combination with other drugs (co-administrations) or administration of phytochemicals as prodrugs or conjugates, particularly when these approaches are supported by nanotechnologies exploiting conjugation strategies with appropriate targeting molecules. These aspects are described for polyphenols and essential oil components, which can improve their loading as prodrugs in nanocarriers, or be part of nanocarriers designed for targeted co-delivery to achieve synergistic anti-glioma or anti-neurodegenerative effects. The use of in vitro models, able to simulate the blood–brain barrier, neurodegeneration or glioma, and useful for optimizing innovative formulations before their in vivo administration via intravenous, oral, or nasal routes, is also summarised. Among the described compounds, quercetin, curcumin, resveratrol, ferulic acid, geraniol, and cinnamaldehyde can be efficaciously formulated to attain brain-targeting characteristics, and may therefore be therapeutically useful against glioma or neurodegenerative diseases.

## 1. Introduction

Nutritional aspects are important in determining human health. A balanced diet including food obtained from plant products and limiting the amounts of red meat, fast foods, saturated fat, sugar, and salt contributes, in fact, to a healthy life [1]. For example, a healthy diet stimulates the growth of beneficial bacteria in the gut microbiota [2], and decreases the risk colorectal cancer [3]. On the other hand, inappropriate nutrition favors an accumulation of DNA damage at the cellular level, thus leading to an increased risk of developing cancer [4]. Again, fruits, vegetables and grains seem able to protect against the development of diabetes and cardiovascular disease [5]. Finally, a balanced diet benefits the central nervous system (CNS). High consumption of fruits, vegetables, and olive oil, with moderate consumption of alcohol (i.e., the Mediterranean diet), lowers the risk of developing cognitive impairment and neurodegenerative diseases [6].

Pharmacologically active phytochemicals, which are bioactive ingredients produced as secondary plant metabolites as growth regulators or repellents to pests, and sunlight contribute to the beneficial effects induced by a balanced diet [7].

The antioxidant and anti-inflammatory properties of polyphenols, one of the major categories of phytochemicals, contribute to these compound beneficial effects by modulating the activation of cytokines and regulating the gene expression of pro-inflammatory molecules [1]. Essential oils are other phytochemicals characterised by antioxidant and anti-inflammatory properties [8], and are potentially beneficial at both peripheral [9,10] and central levels [11,12,13,14].

Cancer cell invasion and metastasis have been hypothesised to originate from a small subpopulation of cancer stem-like cells (CSCs), i.e., relatively slow-cycling quiescent cells. Accordingly, the specific elimination of CSCs appears therefore to be of significant importance for cancer eradication. Unfortunately, CSCs can survive conventional chemotherapy, leading to recurrent disease [15]. Several phytochemicals can target and destroy CSCs [16,17] by inhibiting specific signalling pathways (for example WNT/b-catenin) that confer them high resistance [18]. Therefore, combinational treatments with standard chemotherapy and phytochemicals have been proposed as promising for cancer eradication [15].

The beneficial effects of dietary phytochemicals are usually minimal [19] due to the poor bioavailability and rapid metabolism of compounds [20]. These aspects may limit the use of phytochemicals as potential drugs for acute or long-term therapies against both peripheral and central diseases. Concerning brain diseases, it is known that several phytochemicals can penetrate the CNS from the bloodstream [13,21,22], but their permanence in the brain appears low and poorly adequate for therapeutic proposals [23,24]. Consequently, remarkably high doses of these compounds are generally necessary to induce neuroprotective effects [12].

**Figure 1:**
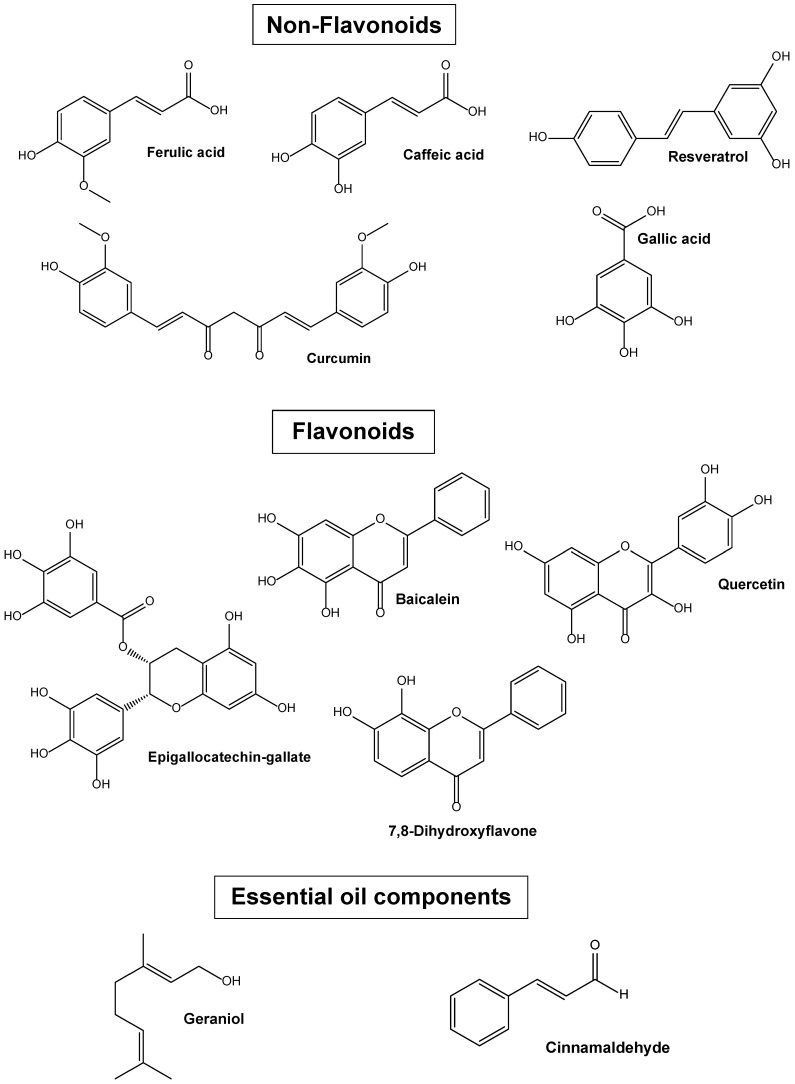
Phytochemicals showing activity in the central nervous system.

Considering the interesting potential therapeutic properties of phytochemicals, several strategies are currently under study to improve their poor permanence in the body. This review summarises the results of studies aiming to individuate innovative strategies to improve the efficacy of phytochemicals in the CNS through exploiting combination therapy with other drugs (co-administrations), or through their administration as prodrugs or conjugates. Particular attention has been paid to nanotechnology formulations supported by conjugation strategies with appropriate targeting molecules [15,25,26,27,28,29,30,31]. The phytochemicals most evaluated in the reviewed studies are polyphenols, classified as non-flavonoids (gallic acid, ferulic acid, caffeic acid, curcumin and resveratrol) or flavonoids (baicalein, 7,8-dihydroxyflavone, epigallocatechin gallate and quercetin) [32], and the main components of essential oils (geraniol and cinnamaldehyde) [31,33]. The chemical structures of these neurotropic phytochemicals are reported in Figure 1. The innovative formulations based on these phytochemicals, detailed herein, are those designed for exploiting their beneficial effects against several brain pathologies (such as schizophrenia, Parkinson’s disease, depression, Alzheimer’s disease, ischemic stroke, cognitive disfunction, neurodegeneration and glioma), either by using in vitro models based on appropriate cell lines, or in vivo animal models. In the following review sections, (i) the cellular mechanisms underlying the neuroprotective or CNS anticancer effects of the selected phytochemicals; (ii) the in vitro cellular models that can be used to evaluate the selected phytochemical’s efficacy against brain CNS diseases, and the ability of phytochemicals to target, when properly formulated, the brain; and (iii) a summary of the main innovative formulations designed to enhance the central effects of the selected phytochemicals will be presented.

## 2. Phytochemicals’ Neuroprotective or Anticancer Mechanisms of Action

### 2.1. Phytochemicals for the Therapy of Neurodegenerative Diseases

It is now generally accepted that although neurodegenerative diseases such as Alzheimer’s disease (AD), Parkinson’s disease (PD), Huntington’s disease (HD), and Amyotrophic lateral sclerosis (ALS) display different features and clinical symptoms, they share some mutual pathogenetic mechanisms [34,35]. These devastating diseases are characterised by a progressive loss of selected neuronal populations, leading to motor dysfunction, cognitive impairment and several disabilities associated with a reduction in patients’ quality of life and premature death. The precise mechanisms underlying neuronal cell death in neurodegenerative diseases still remain unclear. The current view is that several factors contribute to neuronal loss in neurodegenerative diseases. Among these factors, misfolding and erroneous protein aggregation, increased oxidative stress related to the generation of reactive oxygen species (ROS), chronic inflammation, hippocampal adult neurogenesis differentiation/disruption, apoptosis induction, brain blood flow alterations and blood–brain barrier (BBB) disruption, neural and glial cell metabolism changes, neurotransmitter dysregulation, white matter dysfunction, and microbiota and gut–brain axis alterations make up the theoretic framework for neurodegenerative disease development [34,35]. A main reason underlying the current failure of pharmacological treatments to block/slow neurodegenerative disease progression could be the monotherapy approach, which seems inadequate to break the vicious cycle triggered by these factors. Thus, it is conceivable that compounds with a broad spectrum of action blocking multiple disease-causing or disease-progressing pathways could have a higher impact on neuronal loss progression than current classical pharmacological treatments [36,37]. In this context, phytochemicals are emerging as interesting complementary and/or alternative strategies for countering neurodegenerative disease development and progression, and various studies focused on phytochemicals for neurodegenerative disease management have been published. In fact, several phytochemicals display antioxidant properties of positive modulation on neuroprotection, anti-inflammatory properties, and the ability to cover a wide spectrum of targets that are triggered in neurodegenerative disease [35,37,38,39,40]. The mechanisms underlying these properties are multiple, and cannot be described herein. Some data are therefore here reported as pure examples of how phytochemicals might be beneficial in treating neurodegenerative diseases.

Quercetin, curcumin, anthocyanins, and other polyphenolic compounds modulate nuclear factor erythroid 2-related factor 2 (Nrf2) activity by regulating several Nrf2 upstream activators, thus contributing to contrast oxidative stress [41]. The transcription factor Nrf2, in fact, regulates numerous endogenous antioxidant expressions by inducing the transcription of genes containing antioxidant response element (ARE). It has been recently proposed that ROS-dependent excessive oxidative stress could be modulated by some endogenous ROS suppressors, including the NAD + -dependent deacetylase enzyme family called sirtuins (SIRTs). SIRTs are involved in the regulation of mitochondrial gene expression, as well as in neurodegeneration. Phytochemicals reported to enhance SIRTs include ferulic acid, tetrahydrocurcumin, quercetin and resveratrol [42,43]. Alongside its antioxidant activity, the neuroprotective effects of curcumin on AD have been linked to several mechanisms, such as a reduction in β-amyloid (Aβ) accumulation and tau hyperphosphorylation, anti-inflammatory effects, and metal ion-complexing properties [37,44]. Due to its pleiotropic properties, curcumin has been also proposed for the management of brain traumatic injury and PD. Several other flavonoids, including resveratrol and quercetin, have a broad range of neuroprotective properties due to their ability to suppress neuroinflammation and protect neurons, and have a positive effect on cognition. These compounds have therefore been proposed as useful for managing neurodegenerative disease [45]. Quercetin and other flavonoids were found to suppress nitric oxide (NO) production [46]. This is relevant for their potential neuroprotective properties, since the activation of glial cells and, particularly, astrocytes and microglia induces NO overproduction, thereby leading to neuroinflammation and neurodegeneration. Resveratrol’s biological activity also depends on the ability of the compound to regulate various biochemical mediators. For example, in AD models, resveratrol interferes with the formation of Aβ, stabilises microtubules associated with protein function, inhibits inflammatory response, and improves antioxidant activity; in PD models, resveratrol protects dopaminergic neurons from the insults induced by several toxins and, by modulating the extracellular signal-regulated kinases ERK1/2 and ERK5, reduces the dopamine (DA)-induced apoptotic cell death of human dopaminergic neuroblastoma SH-SY5Y cells [47]. Ferulic acid is another phenolic compound that has been historically investigated as a potent ROS scavenger, with therapeutic potential in various chronic diseases including neurodegeneration [48]. Ferulic acid displayed direct effects on neural stem cell proliferation, thus stimulating neurogenesis in vivo. Furthermore, the ability of ferulic acid to mediate the communication between the commensal microbiota and the brain has recently been reported [49]. This aspect is particularly interesting in view of the role assigned to the gut microbiota as a potential target for various chronic diseases, including neurodegenerative diseases [50]. It is also worth noting that oxidative stress, inflammation and immune system alterations have also been involved in other brain disorders such as schizophrenia [51]. Consequently, several phytochemicals have been also investigated for their putative anti-psychotic action in cell culture and animal models of CNS disorders. For example, concerning polyphenols, baicalin seems to have the potential to ameliorate negative symptoms and cognitive impairment in animal models of psychosis, possibly due to its anti-prolyl-oligopeptidase, anti-inflammatory and antioxidant actions [51]. Moreover, quercetin has been reported to ameliorate symptoms of schizophrenia because of its free radical scavenging activity [51]. Despite all these promising properties of phytochemicals, their usage as therapeutics for CNS disorders is restricted, especially due to their limited bioavailability in the brain. Overcoming this limit is therefore critical for the development of phytochemical-based products to manage neurological disorders.

### 2.2. Phytochemicals against Cancer Relapse

Several data suggest that in tumours, CSCs, a subgroup of cancer cells characterised by stem-like properties, can significantly contribute to chemoresistance, cancer relapse, invasiveness and development of metastasis. CSCs indeed appear able to self-renew and differentiate into heterogeneous cancer cell lineages in response to chemotherapeutic agents [52]. The origin and tumourigenic development of CSCs have been suggested to derive from normal cells in the body prematurely losing their capacity to proliferate; this is caused by strongly hostile local conditions. These cells attempt to escape their premature death, which may be imminent, by activating their encrypted dark genome, therefore gaining their progenitor atavistic cell lineage to retrieve survival properties typical of single-cell eukaryotes. These cells, indeed, are characterised by adaptive mechanisms developed over millions of years, which allow them to protect themselves from exposition to hostile life conditions, such as variations in oxygen and nutrients, harmful chemicals, or increased radiation. These adaptive mechanisms do not belong to healthy body cells, which are normally protected in homeostatic compartments. Therefore, under particular local conditions that become hostile for body cells, these adaptive mechanisms can induce the reappearance of unicellular features that lead to the origin of CSCs [53].

In solid tumours, CSCs appear localised in niches characterised by hypoxic microenvironments, and can be recognised as a subpopulation of “colourless” cells, being able to expel typical DNA-binding dyes [15,36]. These hypoxic conditions enhance the intrinsic quiescence of CSCs, and therefore increase their resistance to conventional anticancer drugs designed to rapidly kill proliferating cells [15].

The specific elimination of CSCs appears of crucial importance to eradicate cancers from the body; unfortunately, conventional chemotherapies are unable to kill these cells, whose targeting appears extremely difficult, them being localised in protected niches in solid tumours. Consequently, combinational treatments based on both conventional chemotherapy and drugs able to target and kill the CSCs have been proposed as promising strategies to induce cancer eradication [15,54,55,56].

Specific markers expressed on CSCs’ surface, such as CD133 or CD44, can allow the targeting of this type of cells. These markers are glycoproteins that are essential for stem cell survival and activity, and can be recognised by specific molecules opportunely inserted into nanocarrier systems. For example, CD44 is recognised by hyaluronic acid [56,57]. Aldehyde dehydrogenase 1 (ALDH-1) is another CSC marker associated with cancer progression [58].

Specific signalling pathways, such as Hedgehog, Notch, or WNT/β-catenin, induce transcriptional processes essential for CSCs survival [59,60,61]. A strategy focused on interfering with these signalling pathways can be therefore crucial to compromise the proliferation of CSCs and the increase in tumourtumour growth and invasiveness [15].

The anticancer properties of several phytochemicals (such as resveratrol or curcumin) have been studied from more than 30 years, but only recently have these properties been correlated to their ability to destroy CSCs [16,17]. In particular, curcumin is known to reduce the β-catenin activity in cancer cell lines and the transcription of target genes in the WNT/b-catenin pathway typical of CSCs [18]. Curcumin was also found to selectively target CSCs in human carcinoma cell lines by inducing significant loss of the ALDHA1+ and CD44+ cell populations [62]. These properties have stimulated several investigations into the possible therapeutic properties of curcumin in combination with conventional chemotherapeutic agents [16]. This approach was efficacious against CSCs, with slight or no effect on differentiated health cells [17,63,64].

Similarly, the combination of resveratrol with a dietary supplement associated with anticancer properties induced CSC apoptosis by interfering with the WNT/b-catenin signalling pathway, without displaying the typical side effects produced by conventional chemotherapeutics [65].

The following sections will describe how nanotechnologies can contribute to selectively targeting this type of therapies against glioma.

## 3. Cellular Models to Study In Vitro the Efficacy of Phytochemicals against Brain Diseases, and Their Ability to Target the CNS

Due to the ethical and cost limitations of in vivo animal models, in vitro replaceable cell models, related to the characteristics of organisms, provide a safe, easily applicable and reproducible tool for drug screening. For example, these models allow the evaluation of the compound absorption rate, as well as the investigation of the possible mechanism of action of substances able to interfere with specific pathways involved in CNS disease etiopathogenesis [66].

Cellular models allow us to evaluate the ability of phytochemicals to induce enhancement of cellular defence mechanisms and restoration of stress response signalling, which are important properties used to predict several possible in vivo therapeutic applications of putative drugs [67].

Cellular models may be based on immortalised, tumoral or spontaneously stabilised cell lines, with an intrinsic ability to grow in a monolayer when seeded on semi-permeable membrane supports. In these supports, the cells can express their functions of epithelial physiological barriers, thus representing a useful tool to predict the ability of a compound to permeate biological membranes, or its cellular uptake [68].

Monolayers of brain endothelial cell-based in vitro BBB models include the immortalised human capillary microvascular endothelial cell line hCMEC/D3 and the mouse immortalised brain microvascular endothelial cell line bEnd.3. These cells are tightly coupled by intercellular junctions, called indeed tight junctions, that significantly reduce permeation of ions and large hydrophilic solutes through the paracellular pathway. These BBB cell line models are widely used and well characterised for functional and transport assays, although they do not form a tight barrier, the primary brain microvascular endothelial cells (BMECs) do instead [69]. Usually, the transendothelial electrical resistance (TEER) values of the cell lines are relatively low, and the permeability measured by paracellular markers is significantly high compared to primary BMECs. On the other hand, they are suitable models for large-scale drug transport studies of large molecules based on their receptor expression pattern, surface charge and transcellular properties. As another advantage, the mouse bEnd.3 and human hCMEC/D3 cell lines originate from systematically characterised species, thus providing data translatable to the preclinical and clinical studies. For instance, the mouse bEnd.3 cell line complements the in vivo mouse studies that are widely used in preclinical research, while the human hCMEC/D3 cell line can predict the outcomes of clinical studies [69]. The advantage of these brain endothelial cell lines is also supported by the evidence that human-induced pluripotent stem cells differentiated into brain microvascular endothelial cells (ihBMECs) have superior barrier properties, such as higher TEER values; however, their differentiation process takes a long time and can result in mixed endothelial–epithelial gene expression [70]. Overall, both bEnd.3 and hCMEC/D3 cells express key brain endothelial phenotypic markers, and can discriminate between the passage of tracers of different molecular weights [70]. For these reasons, these cells represent high-throughput in vitro models of brain endothelial barriers to predict the delivery of therapeutics to counter neurodegenerative diseases, (e.g., PD and AD), traumatic brain injury, neuroinflammation processes, and tumoral evolution of gliomas, neuro- and glioblastomas of CNS.

Other immortalised CNS cell lines, originating from human or murine gliomas or neuroblastoma, are used to simulate neurons and glial cells, and cultured as a monoculture or as 2D co-culture or 3D models called organs-on-chips or biochips, which more exactly replicate the in vivo neuronal and glial cells surrounding the BBB [66]. Among them, the rat adrenal pheochromocytoma PC12 cell line is known to synthesize and store DA and sometimes norepinephrine (NA), and is induced to differentiate into a mature sympathetic neuron-like phenotype by the nerve growth factor (NGF). For this reason, the rat PC12 cell line is commonly used in neurobiology, including studies of neurotoxicity, neuroprotection, neurosecretion, neuroinflammation, and synaptogenesis, and as a leading dopaminergic model in molecular neuroscience [66]. Therefore, PC12 cells represent a suitable model to study the pathogenesis of PD and AD disorders and ischemia, also providing the opportunity to measure the production of proinflammatory cytokines, such as tumour necrosis factor (TNF)-α, which underlie the onset of these pathologies [71]. On the other hand, the rat origin and the mandatory NGF-induced neuronal differentiation of PC12 cells causes the results obtained with this cellular model to be confirmed in more translational neuronal cell lines, such as the immortal cell line SH-SY5Y, derived from a human neuroblastoma [66]. SH-SY5Y cells can be differentiated using retinoic acid to a more mature neuron-like phenotype characterised by neuronal markers. Indeed, the use of primary neurons derived from mammalian embryonic CNS tissue is limited, because once terminally differentiated into mature neurons, these cells cannot undergo mitosis and so can no longer be propagated. Transformed neuronal-like cell lines overcome this limitation [72], although the retained tumoral properties of SH-SY5Y cells, including the instability of the proliferation, differentiation, and metabolism, assume several caveats when using them as model in neurobiology, as extensively reported by Kovalevich and colleagues [72]. However, the malignant properties of SH-SY5Y cells are turned to an advantage when used to develop anticancer and antiproliferative drugs against metastatic neuroblastomas. With regard to in vitro cell models of glioblastomas, the tumorigenic rat C6 glioma cell line together with the glioblastoma U87 cell line and the astrocytoma T98 cell line are reported to secrete high levels of well-known invasion-promoting proteins, compared to less invasive cell lines [73]. These cell lines therefore could provide suitable models to understand the behaviour of glioblastoma multiforme, the most common and deadly type of brain tumour, and to test drugs against newly identified targets that promote its extensive invasion into surrounding healthy tissue.

In conclusion, further comprehensive studies on the properties of in vitro CNS cell models along with their in vitro–in vivo correlation with human data are needed to achieve future optimization of the use of these models in drug discovery and clinical development.

## 4. Innovative Formulations Designed to Enhance the Central Effects of Neurotropic Phytochemicals

### 4.1. Innovative Formulations Designed to Act against the Neurodegenerative Diseases

Polyphenols, or the main components of essential oil, often appear able to permeate the brain from the bloodstream and are promising agents against neurodegenerative diseases. On the other hand, their fast elimination from the body compromises their use as therapeutic agents [12,13,20,21,22,23,24]. Co-administration forms and the development of prodrugs designed as conjugates with biocompatible molecules appear promising methods to obtain innovative nanocarriers that are able to cross the BBB and to deliver phytochemicals to the brain, thus strengthening their therapeutic effects. These aspects are described below.

#### 4.1.1. Ferulic Acid in the Design of New Formulations against Brain Diseases

Antioxidant and anti-inflammatory properties characterise ferulic acid, which is currently considered useful for the prevention and therapy of neurodegenerative diseases [22]. Ferulic acid is known for its high absorption rate and ability to cross the BBB [74]; despite this behaviour, fast elimination processes limit its presence at peripheral and central levels of the body, wherein adequate ferulic acid amounts for therapeutic proposals cannot be easily obtained [23,75].

New strategies are therefore necessary to increase the amount and permanence of ferulic acid in the brain upon its administration. Taking into account these aspects, ferulic acid-loaded nanostructured lipid carriers were proposed to alleviate ischemic neural injuries, following their intravenous administration [76]; again, self-microemulsifying systems were prepared to increase the oral delivery of ferulic acid and its hypnotic effects [23], and ferulic acid-loaded solid lipid nanoparticles coated with chitosan were designed as a nasal formulation able to enhance the brain-targeting of ferulic acid, improving its anti-AD potential [77]. An alternative approach has been developed, building on the evidence that neuroinflammation in neurodegenerative diseases induces monocytes and neutrophils to permeate the BBB from the bloodstream [78]. These inflammatory cells were therefore identified as potential carriers of ferulic acid. The integrin receptors expressed on their membranes can, in fact, interact with the RGD peptide (Arg-Gly-Asp), which can be used to decorate the surface of ferulic acid-loaded liposomes, through conjugation of RGD via a succinic spacer to the cholesterol used for the formulation of liposomes. The rationale was that the interaction between RGD and the integrin receptors should induce the phagocytosis of the decorated liposomes by the inflammatory cells, which are capable of being taken up into the brain in response to neuronal inflammatory processes [78]. It has been demonstrated in ex vitro studies that RGD-liposomes loaded with ferulic acid were able to efficiently bind monocytes and neutrophils, thus inducing a greater antioxidant activity than ferulic acid solution in U937 cells (a pro-monocytic model cell line). Moreover, RGD-liposomes intravenously administered to rat models of brain inflammation (by intra-striatal microinjections of human recombinant IL-1β) allowed a ferulic acid brain distribution in the brain up to six times higher than those measures after the administration of ferulic acid solutions or uncoated liposomes [78,79].

**Figure 2:**
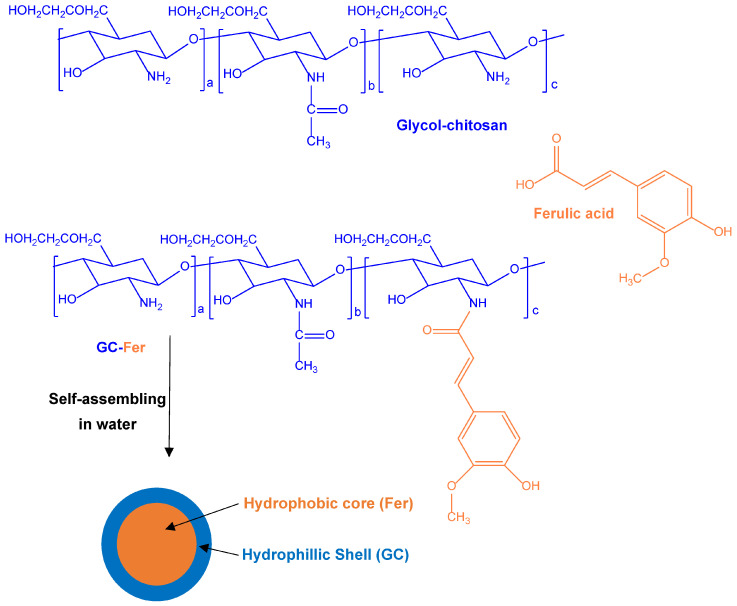
Chemical structures of glycol-chitosan (GC), ferulic acid (Fer), and the polymer obtained by their conjugation (GC-Fer). This polymer was assembled by sonication in aqueous environments, as nanoparticles whose hydrophobic core and hydrophilic shell are constituted by Fer and GC, respectively.

Ferulic acid, because of its neuroprotective effects, has also been proposed for the functional restoration of the traumatically injured spinal cord. In particular, a ferulic acid and chitosan co-delivery strategy to injured sites of ferulic acid and chitosan was designed, considering that also chitosan displays anti-inflammatory and anti-oxidative properties [26]. Ferulic acid was therefore conjugated to glycol–chitosan (GC, chosen as a water-soluble derivative of chitosan) to obtain the GC–ferulic acid amphiphilic polymer (Figure 2). Using sonication in aqueous environments, this polymer was able to self-assemble as GC–ferulic acid nanoparticles, whose hydrophobic core and hydrophilic shell were constituted by ferulic acid and GC, respectively [26]. The GC–ferulic acid nanoparticles were able to protect rat primary neurons from glutamate-induced excitotoxicity. Moreover, after the intravenous administration to rats, the GC–ferulic acid nanoparticles were characterised by a prolonged circulation time, which was necessary to allow effective delivery of both chitosan and ferulic acid to the injured spinal cord sites. Rat models of spinal cord contusion injury evidenced significant recovery in animal locomotor function after receiving the CG–ferulic acid nanoparticles via intravenous administration. Histological analysis of the spinal cord of these rats confirmed the high neuroprotective effects of GC–ferulic acid nanoparticles against spinal cord injury [26].

An alternative strategy to increase the brain-targeting of ferulic acid based on the synthesis of prodrugs recognised by L-type amino acid transporter (LAT1) has also been proposed. The conjugation of ferulic acid with appropriate amino acids allowed the obtainment of substrates transported into retinal pigment epithelia ARPE-19 cells by LAT1 and able to cross the BBB of mice. The presence of an aromatic ring in the promoiety in the amide-based prodrug was essential for the LAT1 transport; moreover, the amide prodrug with the promoiety directly conjugated in the meta-position to ferulic acid, allowing the bioconversion of the prodrugs to ferulic acid in the brain of mice. Analogous ester-based prodrugs did not utilise the transporter for cellular uptake in ARPE-19 cells, and showed higher stability in human plasma with respect to mouse plasma [80]. The prodrugs were taken up more efficiently than ferulic acid (up to 600 times higher) in astrocytes, wherein they evidenced very efficient antioxidant and anti-inflammatory properties [81].

**Figure 3:**
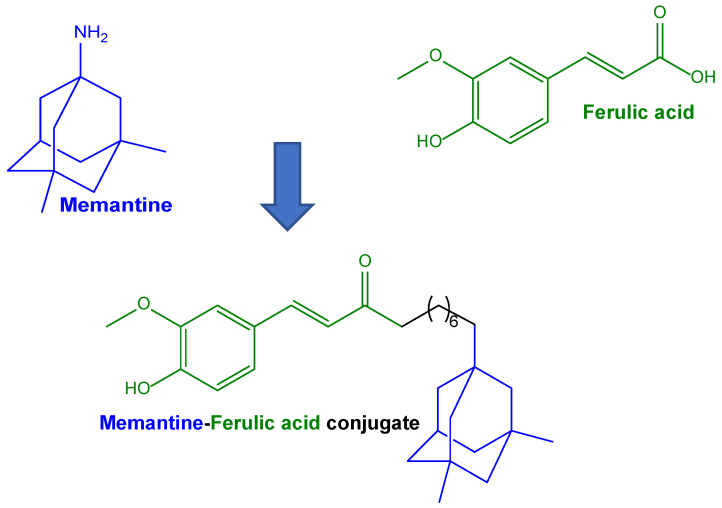
Chemical structures of memantine, ferulic acid, and their conjugate, characterised by multimodal antioxidant properties.

Another type of ferulic acid conjugation was proposed to potentiate the effects of ferulic acid against AD. The over-activation of N-methyl-D-aspartate receptors (NMDAR) can trigger neurotoxic events mediated by Aβ and oxidative stress. These phenomena can be limited by the drug memantine (Figure 3), which is able to limit neurotoxicity mediated by excessive NMDAR activation [82]. Considering these aspects, memantine was conjugated with ferulic acid, with the aim of synergistically targeting and modulating the pathological partnership between oxidative damage, Aβ burden, and hyperfunctioning NMDAR. The derivative obtained with a hexamethylene spacer showed an affinity toward NMDAR similar to that of memantine, thereby evidencing multimodal antioxidant properties in SH-SY5Y cells [82].

The methyl ester derivative of ferulic acid (Fer-Me) is a ferulic acid prodrug able to preserve the antioxidant properties of the parent drug and its anti-inflammatory behaviour, as evaluated in vitro on PC12 cells chosen as a model for neural differentiation [71]. Solid lipid microparticles (SLMs) based on tristearin or stearic acid were loaded with ferulic acid or Fer-Me, evidencing the aptitude of the prodrug to increase loading in SLMs in comparison to ferulic acid. Tristearin-based SLMs were able to increase the dissolution rate of Fer-Me in water, also inducing a control of the release of both ferulic acid and Fer-Me; their intramuscular administration was therefore proposed to counter neuroinflammation related to peripheral neuropathic pain. Stearic acid-based SLMs were able to induce a very fast dissolution of Fer-Me within a few minutes; their nasal administration was therefore proposed with the aim of inducing derivative brain uptake [71].

#### 4.1.2. Caffeic Acid in the Design of New Formulations against Brain Diseases

Caffeic acid (3,4-dihydroxycinnamic acid) is a phenolic acid (non-flavonoid) derived, similarly to ferulic acid, from the hydrolysis of chlorogenic acid. The main source of this compound is food (fruits, grains, vegetables, tea, coffee); after the consumption of dietary substances, it is absorbed in the gastrointestinal tract and may be able to cross the BBB and reach brain cells.

Caffeic acid can potentially prevent several human diseases because of its wide range of pharmacological properties, such as antidiabetic, antihypertensive, antioxidant, anti-inflammatory, anticancer, immunomodulatory and neuroprotective properties [83]. The presence of caffeic acid in human CSF has been described for the first time in a recent study suggesting that phenolic acid can cross the BBB or blood-CSF barrier, exerting a neuroprotective effect with a beneficial influence on brain function during aging [84].

The therapeutic effects of caffeic acid in cancer and neurodegenerative disorders may be synergistic with those of various active agents. A successful therapeutic strategy was achieved by combining phenolic acid with chemotherapeutic cisplatin in a dose ratio of 50:5 mM, which enhanced caspase activity in A2780cisR cells (a cisplatin-resistant cell line). Furthermore, when caffeic acid was combined with metformin, synergistic results were obtained, prompting apoptosis without damage to healthy human fibroblasts, with an anticancer activity specifically toward MS 751 cells (human cervical epidermoid carcinoma cell line) [85].

Some studies are also focused on nanosize-based drug delivery systems as a strategy to enhance the efficacy of caffeic acid therapeutic treatments. In particular, nanoparticles obtained through ion complex formation between caffeic acid-conjugated chitosan and carboxymethyl dextran-b-poly(ethylene glycol)-loaded doxorubicin were tested on CT26 cells (murine colorectal carcinoma cell line). The results revealed this formulation to be a promising vehicle for anticancer drug targeting [86]. Caffeic acid in solid lipid nanoparticles was also tested using an MTT assay; compared to the free drug, it was less toxic to NIH/3T3 normal cells (a fibroblast cell line), but more cytotoxic to H-Ras 5RP7 (an oncogene-transformed fibroblast cell line), thus appearing promising for the treatment of clinical tumours [87].

Šebestík and co-workers prepared bifunctional choline esters of caffeic acid derivatives, combining, in the same molecular entity, the antioxidant properties of phenolic acid with the inhibitory activity of acetylcholinesterase (AChE); the resulting protection against 1-methyl-4-phenylpyridinium ion (MPP+)- or Aβ peptide-induced neuroblastoma SH-SY5Y cell death suggested the neuroprotective properties of these derivatives in PD and AD, respectively [88].

The AChE inhibitory effect was also proved in rats in the study of Akomolafe, wherein a combination of caffeine and caffeic acid was tested in vivo. This result could be of pharmacological and therapeutic interest, especially in the treatment of those neurodegenerative diseases, such as AD, which are characterised by impaired cholinergic function and elevated AChE activity [89].

Mishra and co-authors developed budesonide (BUD)-loaded micelles for the management of ulcerative colitis (UC) in a mouse model [90]. UC is an inflammatory bowel disease (IBD) causing chronic inflammation in a specific portion of the large intestine. BUD is a corticosteroid drug with potent localised anti-inflammatory activity, but its therapeutic efficacy was limited by its low bioavailability due to an extensive first pass metabolism after oral administration. The drug delivery system project involved the preparation of nanosized micelles based on an amphiphilic compound obtained through conjugation of caffeic acid to stearic acid by means of ethylenediamine as a linker, which encapsulated BUD. The use of caffeic acid allowed the enhancement of the aqueous dispersibility of micelles for convenient rectal administration; an in vivo study in a mouse model of UC showed the significant therapeutic efficacy of this formulation. This result is promising considering the importance of the gut microbiota–brain axis and the relevant role of intestinal well-being in neurodegenerative diseases such as PD and AD [91].

#### 4.1.3. Gallic Acid in the Design of New Formulations against Brain Diseases

Gallic acid (3,4,5-trihydroxybenzoic acid) is a natural phenolic acid found in many fruits and medicinal plants such as grapes, berries, wine, and tea, in which it occurs in the form of free acids and as esters, catechin derivatives, and hydrolyzable tannins [92]. In recent years, this phytochemical compound has attracted increasing interest owing to its different pharmacological properties such as antibacterial, antioxidant, anticancer, cardioprotective, gastroprotective, and anti-inflammatory properties [93,94]. For these reasons, gallic acid is applied in the pharmaceutical, cosmetic, food, and dyeing industries [95]. In particular, in the food and cosmetic industry, gallic acid is used as an additive, due to its capacity to inhibit the oxidation and rancidity of oils and fats [96].

Besides these properties, gallic acid has demonstrated important activity in preventing proteins’ misfolding, as well as in reducing the cell toxicity induced by fibrillar protein aggregates; therefore, it has been tested in the inhibition of fibrillar protein deposits that lead to CNS disorders such as AD and PD [92]. In particular, gallic acid inhibits α-synuclein aggregation, preventing the loss of dopaminergic neurons and consequently exhibiting great neuroprotective effects.

Despite this pharmacological activity, gallic acid, due to its high hydrophilicity (LogP ≈ 0.42), is not able to cross the BBB. In the attempt to ameliorate the lipophilicity and, consequently, to permeate the BBB of this phenolic acid, Li Chen and co-authors synthesised a series of derivatives, particularly amide derivatives, with a sheet-like conjugated structure. These derivatives showed in vitro anti- α-synuclein aggregation properties, with an IC_50_ value as low as 0.98 μM, thereby representing good candidates for the treatment of neurodegenerative disease [97].

The antioxidant activity of gallic acid was also exploited by Dan Zhang and co-authors to develop an injectable hydrogel for the treatment of traumatic brain injuries mainly caused by ROS overexpression [98]. Hydrogels were an efficient drug delivery system for in situ CNS applications, with good biocompatibility due to their capacity to mimic the extracellular matrix in the brain [99]. To improve its antioxidant activity, in this research, gallic acid was conjugated with gelatine, a degradation product of collagen characterised by good biocompatibility and low immunogenicity [100]. The conjugation product (GGA) was subsequently linked to oxidised dextran (Odex) to obtain the GGA6Odex hydrogel with excellent injectable, self-healing, antioxidant, and biocompatible properties. The gel has also demonstrated in vitro the ability to protect cells from oxidative damage and, in an in vivo mouse model, the ability to facilitate neurogenesis and promote motor, learning and memory abilities, thus representing a promising biomaterial for tissue regenerative medicine, including the treatment of traumatic brain injury.

#### 4.1.4. Resveratrol in the Design of New Formulations against Brain Diseases

The literature reports extensive evidence of applications of resveratrol, a non-flavonoid polyphenolic molecule, as a cardioprotective [101] or anticancer agent [102]. However, recent interest in its neuroprotective activity and employment in neurological diseases, such as AD, PD, glioblastoma and other brain tumours, multiple sclerosis, depression and anxiety, and epilepsy, is growing [103,104,105,106]. The molecule has largely been studied for its efficacy against oxidation and inflammation, both phenomena largely involved in the occurrence of neurological disease. In the case of AD, resveratrol interrupts the mechanism by which ROS promote Aβ production, which in turn induces the occurrence of ROS. Resveratrol inhibits the accumulation of ROS [107], stimulates HO-1 activity, and activates SIRT1-mediated inhibition of hydrogen peroxide production. In PD, resveratrol inhibits ROS produced by hippocampal cells by activating AMPK, suppresses COX-2 and lipid peroxidation and increases the production of antioxidant enzymes [104,108]. The interest in this molecule is also related to its large availability in the diet, as it is a component of red grapes and wine, chocolate, blueberries, cranberries, and peanuts. It is classified as a Biopharmaceutics Classification System (BCS) class II drug, with good permeation but low bioavailability due to low solubility, and also fast metabolism. However, its permeation of the BBB is modest, as the Papp was found to be lower than 10 × 10^−6^ cm/s, about ten times lower than for caffeine [109]. Nanocarriers have therefore been developed to improve its BBB permeation thanks to nanoparticles coating the surface, especially with polysorbate 80, or decoration with targeting ligands. Among these, resveratrol delivery to the CNS has been improved by loading into poly(lactic-co-glycolide) (PLGA) nanoparticles (NPs) decorated with lactoferrin (Lf), a cation iron-binding glycoprotein of the transferrin (Tf) family, whose receptors are highly expressed in the brain in the case of PD [110]. Intracellular ROS production was assessed in SH-SY5Y cells, and a significant reduction in ROS after H_2_O_2_ treatment was observed in the presence of Lf-resveratrol-PLGA-NPs in comparison to the cells pretreated with free resveratrol or with resveratrol-PLGA-NPs. Significantly higher levels of neuroprotection were also observed in vivo after intravenous injection in an MPTP-induced PD mice model. Similarly, a ligand peptide of low-density lipoprotein receptor (LDLR) was conjugated with polylactic acid (PLA)-coated mesoporous silica nanoparticles. The authors demonstrated that these systems were useful for improving the BBB passage of resveratrol and its selective release to reduce inflammation caused by ROS overproduction. This selective effect was due to the higher degradation rate of PLA, which is responsible for the control of drug release, in the presence of ROS [111].

As it has been reported in a complete and recent review [112], the proposed modifications of the phenolic hydroxyls of resveratrol to obtain prodrugs are quite numerous. These modifications aimed either to increase the bioavailability of resveratrol by improving its chemical stability, stability in blood, and solubility in water, or to modulate its pharmacological activity. In this last case, conjugations result in increased antioxidant activity or in higher cytotoxicity, which is useful for better anticancer efficacy [112].

Natural resveratrol derivatives such as the monomethylated pinostilbene and dimethylated pterostilbene have higher neuroprotective efficacy than resveratrol [113,114,115,116]. Besides them, piceid, a naturally occurring glycoside precursor of resveratrol has been found to be endowed with higher scavenging activity than resveratrol and higher stability towards enzymatic oxidation. It is active as an antioxidant agent in SH-SY5Y cells and can protect them from DA-induced apoptosis [117]. These findings inspired the design of the alkylated resveratrol derivatives, prodrugs and metabolites described in the paper of Penalver et al. [118]. At 1–10 µM concentration, all the several methylated and butylated derivatives tested showed positive antioxidant activity, better than that of resveratrol, in SH-SY5Y neuroblastoma cells treated with H_2_O_2_. Some prodrugs (3,5-diglucosyl-resveratrol, piceid octanoate and three butylated resveratrol glucosylated prodrugs) were studied in a zebrafish model of pentylentetrazole-induced epilepsy, displaying anticonvulsant properties, particularly relevant for piceid octanoate. In line with the anti-inflammatory activity of short- and medium-chain fatty acids, octanoyl piceid derivative showed higher anti-inflammation and neuroprotection than resveratrol. Moreover, the derivative glycosylation meant it was necessary to reduce the toxicity in zebrafish model, as demonstrated by the higher toxicity was of non-glycosylated alkyl derivatives [118].

Silyl derivatives of resveratrol were more recently evaluated by the same research group, with further preparation of their prodrugs with acyl-, glucosyl- and carbamoyl- functions [119]. In particular, disilyl derivatives containing triethylsilyl (TES) and tri-isopropylsilyl (TIPS) groups showed better neuroprotective capacity and anti-inflammatory activity than resveratrol. Among the prodrugs, the 3,5-triethylsilyl-4′-(6″-octanoylglucopyranosyl) derivatization resulted in a reduced motor deficit in mice models of HD. A study on mice with an induced chronic experimental autoimmune encephalomyelitis (EAE) model of multiple sclerosis showed the positive therapeutic effect of the studied derivative; it was able to reduce the clinical progression of the disease in contrast with resveratrol.

An even more recent study on resveratrol derivatives, based on resveratrol cyclic analogues, focused especially on the anticancer effects of the new structures; however, it demonstrated their ability to inhibit tau protein phosphorylation, which is commonly involved in neurodegenerative disorders [120].

A different approach to improve the biopharmaceutic and efficacy aspects of resveratrol involves the association with piperine [121]. Piperine is an alkaloid present in black pepper, with many biological effects such as antioxidant, anti-inflammatory, and immunomodulatory activity. Piperine is known for its ability to improve the bioavailability of polyphenols such as epigallocatechin-gallate, curcumin and resveratrol, through inactivation of cytochrome P450 and other enzymes involved in their metabolism [121,122]. Piperin and resveratrol co-supplementation (10 mg/kg), when compared to resveratrol administration, induced more than ten times the enhancement of maximum serum resveratrol levels and an AUC increase of 229% in mice [123]. The study of Wightman and coworkers [121] demonstrated that the effect of co-supplementation of piperine with resveratrol could affect not only the polyphenol bioavailability but also its efficacy, measured as cerebral blood flow (CBF) and cognitive function. The study enrolled 23 healthy adults and demonstrated that although CBF was improved by the combined treatment, no effect on cognition, mood, blood pressure or heart rate was observed. The results obtained were explained by the potentiating activity of piperine on the vasorelaxant properties of resveratrol, possibly through its thermogenic properties, specifically in neural tissues.

#### 4.1.5. Curcumin in the Design of New Formulations against Brain Diseases

**Figure 4:**
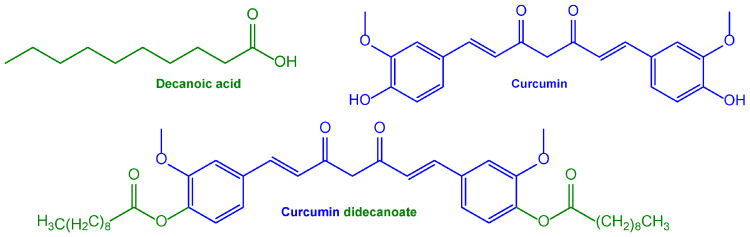
Chemical structures of decanoic acid, curcumin and the prodrug curcumin didecanoate, obtained by their ester conjugation.

The ability to induce anti-Aβ and anti-tau hyperphosphorylation properties, together with antioxidant and anti-inflammatory activities, indicates that curcumin is a promising compound for the treatment of AD. On the other hand, the potential therapeutic applications of this compound are limited by its water insolubility, its very poor oral bioavailability, and its rapid systemic elimination, with consequent limited presence in the brain [124]. For these reasons, curcumin has been proposed not only for its encapsulation in nanocarriers that are able to permeate into the brain, but also as an Aβ-targeting agent decorating the nanocarriers’ surfaces [125,126]. Moreover, insoluble prodrugs were proposed for the formulation of nanosuspensions suitable for intramuscular injectability to obtain a systemic sustained delivery of curcumin. For example, this strategy was achieved with the prodrug of curcumin, obtained by its ester conjugation with decanoic acid (curcumin didecanoate, Figure 4) [127].

Concerning curcumin’s encapsulation in nanocarriers, its loading was proposed in PLGA nanoparticles decorated on their surface with a targeting peptide (Tet-1 peptide, a 12-amino acid peptide, HLNILSTLWKYR) able to specifically interact with motor neurons and induce retrograde delivery in neuronal cells [125]. These nanoparticles appeared able to maintain the antioxidant and the anti-amyloid activities of curcumin without evidencing significant toxicity on LAG cell lines (mouse fibroblast like connective tissue). Moreover, the decorated nanoparticles efficiently increased their uptake in GI-1 glioma cells, in comparison to the non-targeted nanocarriers. These preliminary data suggest that curcumin loaded in the polymeric nanoparticles decorated on their surface with the Tet-1 peptide may be used for the treatment of AD [125].

**Figure 5:**
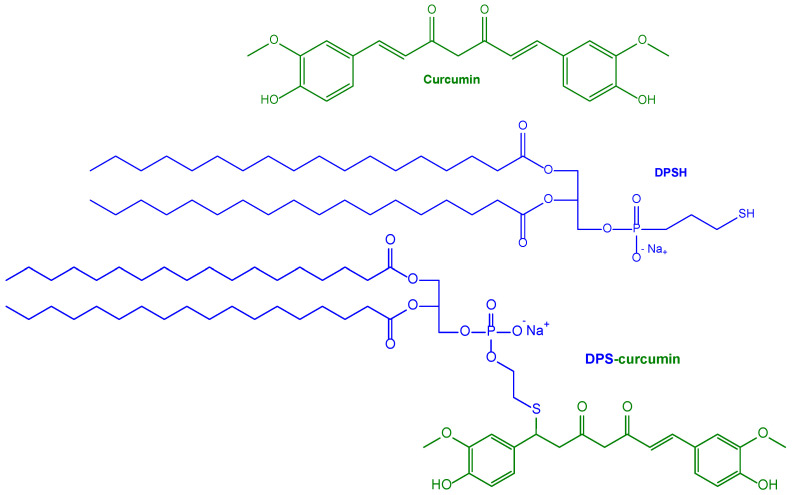
Chemical structures of curcumin, dipalmitoyl-sn-glycero-3-phosphothioethanol (DPSH) and their conjugate DPS-curcumin.

The ability of curcumin to bind Aβ deposits in vivo stimulated the design of nanoliposomes characterised by high amyloid affinity and related targeting properties [126]. In particular, the liposomes were obtained with 1,2-dipalmitoyl-sn-glycerol-3-phosphatidylcholine and cholesterol, in the presence of 1,2-dipalmitoyl-sn-glycero-3-phosphothioethanol (DPSH) conjugated to curcumin (DPS-curcumin, Figure 5), allowing us to obtain small unilamellar vescicles (SUV) with curcumin exposed at their surface [126]. These liposomes did not induce toxicity in HEK cells (from human embryonic kidney), control SH-SY5Y cells and hAPP SH-SY5Y cells, instead stably overexpressing the human APP gene causing familial AD. Moreover, the liposomes were able to partially prevent Aβ-induced cell death and down-regulate the secretion of Aβ peptide in hAPP SH-SY5Y cells, as performed by free curcumin [126]. Finally, the decorated liposomes evidenced a high affinity for Aβ deposits in the post-mortem brain tissue of a transgenic mouse AD model (APPxPS1 mice), and also the ability to specifically stain Aβ deposits in vivo [126].

Considering these aspects, further developments were designed to obtain multifunctional nanoliposomes able to target Aβ deposits after BBB permeation. The following requirements were therefore considered: (i) the blood circulation time suitable for BBB permeation; (ii) decoration of their surface with a BBB transport mediator; and (iii) absence of interferences between amyloid-targeting and BBB transport efficiencies [128].

**Figure 6:**
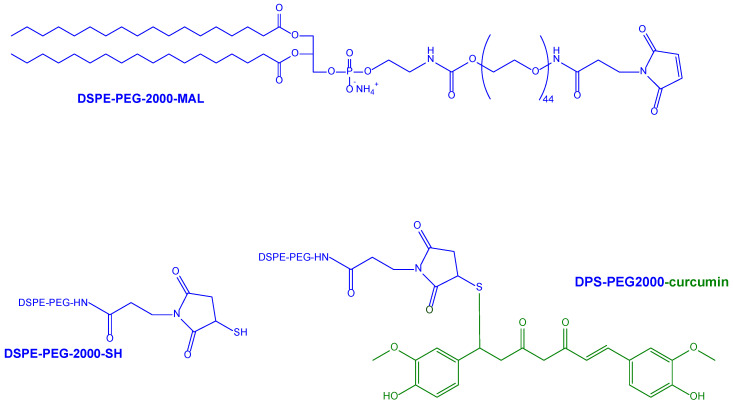
Chemical structures of 1,2-distearoyl-sn-glycero-3-phosphoethanolamine-N-maleimide (polyethylene glycol)—2000 (ammonium salt) (DSPE-PEG2000-MAL), and its thiol derivative (DSPE-PEG2000-SH), which was used for the synthesis of DPS-PEG2000-curcumin.

A polyethylene glycol (PEG) coating was provided for the stealth properties of the nanoliposomes, and also used as an anchoring point for the anti-Transferrin (anti-Tf) antibody, designed as BBB-targeting ligand. Moreover, the curcumin decoration on the nanoliposome surface was placed at a distance from the vesicle bilayer to avoid masking phenomena caused by the PEG coating. In this aim, a new curcumin–lipid derivative (DPS-PEG2000-curcumin) was synthesised, where a PEG spacer was inserted between the lipid and curcumin, conjugated in the presence of a maleimide (MAL) moiety (Figure 6) [128].

High affinity for the amyloid deposits, on post-mortem brain samples of AD patients, was evidenced for these multifunctional nanoliposomes that induce curcumin brain intake, as suggested by a BBB in vitro model (hCMEC/D3 cells). These nanocarriers were therefore proposed as both therapeutic formulations for AD and imaging agents for amyloid deposits in the brain [128].

Multifunctional liposomes were also designed for the co-delivery of curcumin together with the nerve growth factor (NGF) that is known to improve the survival and differentiation of neurons in the hippocampus [129]. In this case, the multifunctional liposomes incorporated cardiolipin in their bilayer structure to induce Aβ targeting properties; furthermore, their surfaces were decorated with wheat germ agglutinin (WGA) anchored to a PEG spacer, to confer the ability to cross the BBB [130]. The liposomes demonstrated reasonable biocompatibility with normal BBB cells, represented by human brain-microvascular endothelial cells (HBMECs), human astrocytes (HAs) and human brain vascular pericytes (HBVPs). The presence of WGA in the liposomes allowed the permeation of the curcumin and NGF across an in vitro BBB model to increase (HBMEC/HA co-cultured cells); moreover, the multifunctional liposomes were able to improve the survival of Aβ-treated SK-N-MC cells (human neuroblastoma cell line) [130].

Loaded curcumin PLGA nanoparticles were provided with GSH (glutathione) functionalization on their surface to increase the neuronal internalization of the formulation. A PEG spacer was inserted between the PLGA polymer and a GSH that was conjugated in the presence of a MAL moiety. The ability of these polymeric decorated nanoparticles to enhance curcumin neuronal internalization was proved in SK-N-SH cells, a human neuroblastoma cell line [131].

The design of a new formulation for the co-delivery of curcumin and the Aβ generation inhibitor S1 (PQVGHL peptide) in the brain was proposed in the form of PLGA nanoparticles loaded with these two compounds and decorated on their surface with the brain-targeting peptide CRT (cyclic CRTIGPSVC peptide) through conjugation to a PEG spacer. The CRT peptide is an iron-mimicking peptide that targets the transferrin receptor (TfR), allowing us to improve BBB’s penetration of the polymeric nanocarrier [132]. These nanoparticles did not demonstrate a significant cytotoxic effect on neuroblastoma SH-SY5Y cells; moreover, the CRT peptide increased their permeation across an in vitro BBB model constituted by brain microvascular bEnd.3 cells, and after its intravenous administration to mice, the distribution of the functionalised nanoparticles in the brain was higher than that of the PLGA nanocarriers without CRT [132]. Accordingly, the CRT-decorated nanoparticles significantly improved the spatial memory and recognition in transgenic AD mice, causing the Aβ and inflammatory factor levels in their brains to reduce [132].

Other functionalised nanocarriers for curcumin were obtained using PLGA-PEG polymeric blocks conjugated with B6 peptide. These blocks were formulated as curcumin-loaded nanoparticles using the emulsion–solvent evaporation method [133]. The B6 peptide (CGHKAKGPRK) decorating the surface of the nanoparticles was designed to enhance drug delivery into the CNS, it being able to target the TfR as a substitute for the Tf protein [134]. In comparison to free curcumin, these functionalised polymeric nanoparticles sensibly increased the cellular uptake in HT22 cells (an immortalised mouse hippocampal cell line); moreover, the functionalised nanoparticles intraperitoneally injected into APP/PS1 transgenic mice (chosen as an in vivo AD model) were significantly more efficacious than native curcumin in improving the animals’ spatial learning and memory capability. Moreover, ex vivo assays demonstrated that these nanocarriers can reduce hippocampal Aβ formation and deposit and tau hyperphosphorylation [133].

Pepe and collaborators studied micellar nanoparticles based on an amphiphilic conjugate of hyaluronic acid with palmitic acid for the delivery of curcumin proposed for the treatment of HD [135]. The micelles increased curcumin’s permeability in vitro on a striatal-derived immortalised cell line expressing mutant huntingtin (STHdh^111/111^), reducing their susceptibility to apoptosis without showing any cytotoxicity effects [135].

Barzegarzadeh and co-workers prepared and characterised in vivo the conjugate of curcumin with linoleic acid [136]. The conjugate was intracerebroventricularly injected for 5 days in male Wistar rats pretreated with ethidium bromide for multiple sclerosis induction. The results demonstrated that the system improved spatial memory in a rat model of multiple sclerosis due to the strong antioxidant effects [136].

As a drug delivery system to enhance solubility, permeability through biological membranes, and the bioavailability of lipophilic drugs, microemulsions (ME) have attracted interest as suitable carriers for several routes of administration, including intranasal administration. Surface-modified ME encapsulating curcumin were synthetized based on a modified Pluronic F127^®^ as surfactant and oleic acid as the oil phase. Firstly, the terminal hydroxyl groups of the surfactant were carboxylated, and subsequently, the carboxylic group on modified Pluronic was conjugated by a carbodiimide reaction to the amino groups of KLVFF (KLVFF-Cur-ME), a peptide for specific binding to Aβ fibrils. It has been hypothesised that bifunctional ME act as an inhibitor of Aβ aggregation, due to the presence of curcumin and KLVFF, and as a targeting agent thanks to the KLVFF peptide. The encapsulation into the ME improved the solubility and the release profile of curcumin. The results from the ex vivo permeation study suggested the successful diffusion of KLVFF-Cur-ME through the porcine nasal mucosa, and did not show nasal ciliotoxicity, suggesting the potential use of this system for nose-to-brain delivery of curcumin [137].

#### 4.1.6. Dihydroxyflavone in the Design of New Formulations against Brain Diseases

**Figure 7:**
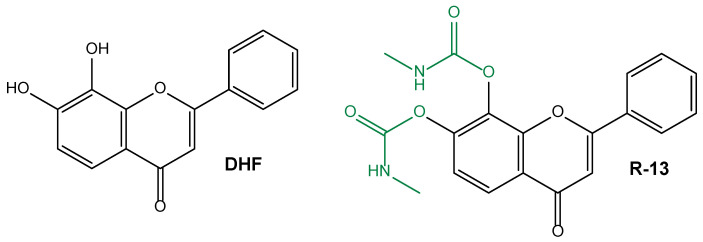
Chemical structures 7,8-dihydroxyflavone (DHF) and its prodrug R-13.

The brain-derived neurotrophic factor (BDNF) is a neurotrophin that contributes to regulating neuronal development, differentiation, and survival via Trk receptors [138]. In brains affected by AD, BDNF expression is poor [139], and its administration appears protective against this pathology [140]. 7,8-dihydroxyflavone (DHF) was identified as a BDNF mimetic compound, being able to specifically interact as an agonist with the TrkB receptor, mimicking the physiological actions of BDNF [141]. DHF is able to cross the BBB [142] and, therefore, it is considered efficacious and safe for chronic and oral treatment of AD. On the other hand, similarly to BDNF, DHF is characterised by poor oral bioavailability and a modest pharmacokinetic profile, showing short half-life values [138]. A prodrug approach was proposed to overcome these problems, and a new DHF derivative, named R13 (Figure 7), was identified as able to significantly increase the oral bioavailability of DHF and its half-life. R13 was indeed related to a DHF prolonged release in the bloodstream of 5XFAD transgenic mice, chosen as in vivo model of neurodegeneration induced by intraneuronal Aβ and the formation of amyloid plaque, which is typical of AD [138]. The 5XFAD mice chronically treated with R13 evidenced activation of TrkB signalling, which prevented Aβ deposition, prevented the loss of hippocampal synapses, and reduced memory deficits in a dose-dependent manner [138]. Very recently, oral administration of R13 to mice affected by peripheral nerve injury was found to induce axon regeneration via TrkB signalling [143].

#### 4.1.7. Epigallocatechin-Gallate in the Design of New Formulations against Brain Diseases

(-)-Epigallocatechin gallate (EGCG) is an ester of (-)-epigallocatechin and gallic acid, constituting one of the main catechins in green and black tea. EGCG is known for its beneficial effects on cognitive functions and against oxidative damage [144], so it appears promising as a potential drug for the treatment and prevention of neurodegenerative diseases. This compound is indeed able to permeate the brain across the BBB, producing, together with its metabolites, antioxidant effects and the prevention of cognitive dysfunction [144]. Moreover, EGCG seems able to promote the regeneration of neurons in animal models of PD, in a manner similar to resveratrol [145]. Despite these interesting properties, EGCG suffers from very poor oral bioavailability and a relatively short half-life in vivo; moreover, in view of its potential important activity in the CNS, its ability to cross the BBB needs to be enhanced [146,147]. The possibility that the co-administration of vitamin E and quercetin or EGCG can favour the accumulation of the two last compounds in the brain has been evaluated [146]. The results of this study suggested that vitamin E does not affect the permeation of EGCG across the BBB, whereas it is able to promote quercetin’s accumulation in the CNS [146].

Recently, liposomes were proposed as carriers for the co-delivery of resveratrol and EGCG in the CNS. In particular, the liposomes were designed to cross the BBB by decorating their surface with leptin (Lep), able to induce transcytosis processes via Lep receptor (LepR) interaction [148]. Moreover, considering that LepR are expressed by neurons [149], the Lep-decorated liposomes were considered potentially useful in facilitating the diffusion of the drugs into degenerated neurons, such as those related to PD [147]. An in vitro model of the BBB based on human astrocytes (HAs), human brain vascular pericytes (HBVPs), and human brain microvascular endothelial cells (HBMECs) was used to demonstrate that the permeation of Lep-decorated liposomes was higher than that of non-decorated liposomes [147]. Moreover, an in vitro neurodegenerative model was obtained with SH-SY5Y cells injured with MPP+ [6], and used to demonstrate that decorated liposomes loaded with resveratrol and EGCG induced their cellular uptake via LepR expressed on SH-SY5Y cells, with consequent cell viability enhancement [147]. The Lep-decorated liposomes loaded with resveratrol and EGCG were therefore proposed as a promising formulation for the therapy of PD [147].

#### 4.1.8. Baicalein Involved in the Design of New Formulations against Brain Diseases

Baicalein and baicalin are natural flavones present in the dried roots of *Scutellaria baicalensis* Georgi. This flowering plant is indigenous to Asia and East Europe and it is widely used in Chinese traditional medicine. Baicalein and its glucuronide, baicalin, possess many pharmacological activities, such as antioxidant, anti-inflammatory, antibacterial, antiviral, antitumour, and neuroprotective effects [150]. The beneficial effects on the CNS are attributed to baicalein, since after oral administration, baicalin is hydrolysed in the gastrointestinal tract by β-D-glucosidases into baicalein. Furthermore, baicalin does not cross the BBB. For these reasons, baicalin is considered a hydrophilic prodrug of baicalein [151].

Baicalein inhibits the activity of protease prolyl oligopeptidases expressed in the CNS in a concentration-dependent manner, and is involved in neuropsychiatric (e.g., schizophrenia and bipolar affective disorder) and neurodegenerative diseases [152].

Baicalein can act as a neuroprotective agent in PD, because it slowed the progression of the disease in an animal model of the pathology (i.e., acrolein-induced neurodegeneration) by inhibiting oxidative stress, protein conjugation, and neuroinflammation [4]. Its anti-neuroinflammatory properties have been associated with the inhibition of the expression of inducible nitric oxide synthase, the production of NO and the nuclear factor-ḳB-signalling pathways in glial cells [150]. The involvement of baicalein in the treatment of PD has been also linked to its ability to restore DA content, neurons, mitochondrial membrane potential, and mitochondrial autophagy protein levels [153].

Baicalein and its oxidised form, baicalein quinone, could also be useful for the treatment of AD; they have been demonstrated to inhibit the aggregation of Aβ and α-synuclein through the formation of a Schiff base [150]. In mice, baicalein reversed the pathways involved in Aβ-induced cognitive impairment and dementia [154]. Baicalein can chelate iron and block the neurodegenerative mechanism linked to ferroptosis, which is common to PD and AD. Baicalein may promote neurogenesis and neural cell differentiation, and has also displayed anti-apoptotic effects [150].

Baicalein also displayed an anti-depressant effects in vitro and in vivo due to its ability to inhibit apoptosis and decrease the release of lactate dehydrogenase; this effect is strongly increased by encapsulation of baicalein in solid lipid nanoparticles, targeted for their high affinity to brain neutrophils [155]. In addition to these nanosystems, new baicalein derivatives were synthesised by binding with amino acids to improve their neuroprotective action [156]. Instead, no conjugates and prodrugs of baicalein were developed.

#### 4.1.9. Quercetin Involved in the Design of New Formulations against Brain Diseases

Quercetin is among the most prevalent flavonoids in the human diet because it is present in many fruits (cranberry, blueberry, cherry, and apple) and vegetables (onion, chili pepper, fennel leaves, lettuce, and spinach) [157,158]. Several in vitro and in vivo studies have suggested the potential pharmacological effects of quercetin such as antioxidant, anti-inflammatory, anti-obesity, antidiabetic, antihypertensive, anticancer, antimicrobial, antiviral, and immunostimulant activities [158]. Quercetin is also a neuroprotective molecule against neurodegenerative and cerebrovascular diseases [159], thanks to (i) its direct (ROS scavenging and metal chelation) or indirect (modulation of antioxidant enzyme activity) antioxidant capacity; and (ii) its ability to inhibit different protein and lipid kinases and to modulate the intracellular signalling, transcription factors and sirtuins [159]. Nevertheless, the therapeutic use of quercetin is limited and debated: it is difficult to attribute the neurological properties to quercetin or its metabolites [159,160]. In fact, quercetin has low and fluctuating oral bioavailability, which is linked to the kind of food or supplement eaten, its low water solubility, and the fact that it undergoes significant intestinal and hepatic metabolism [2,3]. Quercetin metabolites (isorhamnetin, quercetin-3-*O*-glucuronide and tamarixetin) appear quickly in the blood [160]. In addition to its short blood half-life, the brain’s quercetin concentration is low in vivo due to its limited BBB permeability [159]. For this reason, the development of pharmaceutical formulations is required to exploit the therapeutic potential of quercetin. Different strategies were evaluated for this purpose: (i) the cyclodextrin inclusion complex [161] and injectable solid dispersions [160] for increasing the water solubility and stability; (ii) different solid lipid nanoparticles [162,163]; (iii) liposomes conjugated with phosphatidic acid and targeted with apolipoprotein E [25]; (iv) exosomes targeted with a monoclonal antibodies [164]; (v) co-administration with α-tocopherol [146]; (vi) water-soluble conjugates with hyaluronic acid, targeted with a penetrating polypeptide [165]; (vii) quercetin-conjugated superparamagnetic iron oxide nanoparticles [166,167,168,169]; and quercetin-conjugated iron oxide –β-cyclodextrin nanoparticles [170] for improving the oral and/or brain bioavailability of quercetin.

In particular, when orally co-administered in combination with quercetin to rats, α-tocopherol (Vitamin E) resulted in an increase in the brain’s concentration of quercetin, without any pro-oxidant or cytotoxic effects. It was assumed that α-tocopherol promoted quercetin transport across the BBB by modulating the flux and efflux mechanisms [146].

Cen and co-workers found that quercetin, conjugated with hyaluronic acid and targeted with SS31, a penetrating polypeptide, displayed an increased water solubility and BBB permeability, as well as good neuroprotective activity. Hyaluronic acid was important for targeting quercetin release in the area of the ischemic lesion, wherein the hyaluronidases and CD44 receptors were highly expressed. Thanks to SS31, quercetin was able to reduce oxidative stress in vitro and in vivo by acting on the mitochondria of damaged neurons [165].

Ebrahimpour and collaborators studied the effects of quercetin conjugated with dextran-coated superparamagnetic iron oxide nanoparticles (QDSPIONs) on memory impairment in diabetic rats [166]. After 35 days of treatment by gavage, the conjugated quercetin showed higher efficacy in improving learning and memory than free quercetin, due to the reduction in hyperglycemia as well as increased blood stability and brain bioavailability. Both free and conjugated quercetin were also able to maintain the stability of the body weight of diabetic rats [166]. More recently, the neuroprotective effect of QDSPIONs was attributed to the modulation of the miRNAs/NF-κB pathway in the hippocampus of diabetic rats, and in particular to their activity on the NF-κB-dependent neuroinflammatory pathway [167].

The ability of superparamagnetic iron oxide nanoparticles to enhance the brain concentration of quercetin in vivo was also demonstrated by Enteshari Najafabadi and colleagues [168]. They found higher levels of quercetin both in the brain and plasma, after oral administration of QDSPIONs by gavage, than after free quercetin administration using the same method. These results were related to the prolonged blood circulation of quercetin thanks to QDSPIONs, and not to the ability of iron nanoparticles to cross the BBB [168].

Yarjanli and co-authors evaluated the in vitro activity of quercetin conjugated in QDSPIONs on PC12 cells as a neuronal cellular model [169]. Although the conjugation reduced the anti-radical activity of quercetin, QDSPIONs protected PC12 cells against H_2_O_2_ cytotoxicity, showing similar antioxidant, anti-inflammatory, and antiapoptotic effects, but less toxicity than free quercetin [169]. Moreover, quercetin as metal chelator may reduce the neurotoxicity of superparamagnetic iron oxide nanoparticles in vivo, and contribute to the treatment of diseases characterised by brain iron dysregulation, such as AD, PD and stroke [171].

Hashemian and co-workers studied the antiepileptogenic effects of quercetin loaded into superparamagnetic iron oxide nanoparticles coated with β-cyclodextrin and Pluronic F68 [170]. In vivo experiments in an animal model of seizure demonstrated that after intraperitoneal injection, quercetin-loaded nanoparticles reduced seizure behavioural signs and the loss of neurons in the hippocampus, thereby ameliorating hippocampal astrocyte activation [170]. This anticonvulsant effect was better than that of free quercetin, presumably because of the small size of the nanoparticles (<50 nm) and the enhancement of the brain bioavailability of quercetin [170].

#### 4.1.10. Genistein in the Design of New Formulations against Brain Diseases

Genistein is obtained from soy beans. In addition to antioxidant, anti-inflammatory and anticancer properties [172,173], this compound has also oestrogen-like activity, and for this reason, it is used in the treatment of the symptoms of menopause, particularly to contrast osteoporosis [174,175]. Alongside these therapeutic activities, genistein is also efficient as a neuroprotective molecule, as demonstrated in a study on animal models of AD, in which dietary intake of genistein improved mice’s spatial learning and memory [176].

To ameliorate the delivery of this isoflavone to the CNS, Duro-Castano et al. demonstrated the feasibility of loading genistein onto targeted multimodal polypeptide-based nanoconjugates as nanocarriers, in order to obtain neuroprotection and neurotrophic effects. In particular, genistein was loaded with high efficacy onto polyglutamic acid (PGA)-based globular structures, able to form sphere-like cross-linked self-assembled star-shaped (St-Cl) structures, which were revealed to be nanocarriers with low toxicity and prolonged blood circulation times to maximise passive targeting [177]. To enhance the ability of genistein to cross the BBB, this nanocarrier was modified after polymerization with neuroprotective propargylamine (Pr) residues, together with Angiopep-2 (ANG), a peptide ligand specific to lipoprotein receptor-related protein 1, thus obtaining a genistein-carrying polypeptide (St-Cl-Pr-ANG) nanoconjugate, which is a promising candidate for the treatment of early stage AD [178].

#### 4.1.11. Geraniol in the Design of New Formulations against Brain Diseases

Geraniol is known to promote dopaminergic neuron survival by increasing neurotrophic factors, reducing apoptotic marker expression, and leading to the production of antioxidant enzymes. These properties indicate geraniol as a potential candidate for the treatment of PD [11,12]. Geraniol can penetrate the CNS from the bloodstream, but its short half-life limits its use in long term therapies through conventional administration routes [11,21]. Geraniol can be encapsulated in polymeric or solid lipid nanoparticles, but freeze-drying processes induce their loss from nanoparticulate systems, due to a relatively high volatility [33]. Considering that ursodeoxycholic acid (UDCA) is effective in rescuing mitochondrial function in PD patients [179], an ester conjugate of geraniol and UDCA (GER-UDCA) was synthesised and demonstrated to be a prodrug of both these compounds [33]. GER-UDCA was efficiently loaded in solid lipid nanoparticles, allowing a sensible increase in its dissolution rate in aqueous environments. Nasal administration of GER-UDCA-loaded nanoparticles allowed direct nose-to-brain delivery of the prodrug [33].

As an alternative to prodrug synthesis, in order to increase the bioavailability of geraniol in the CNS, an emulsified formulation of geraniol was designed by using the amphiphilic polymer chitosan oleate (CS-OA) as a surfactant to combine the mucoadhesive and absorptive enhancer properties of chitosan with stabilization effects on the oil dispersion [180]. The oral administration of this formulation allowed a sensible increase in the bioavailability of geraniol, and in its aptitude for permeating the CNS from the bloodstream. Moreover, nasal administration of the emulsified formulation induced the uptake of relevant amounts of geraniol to the CNS [180].

Finally, nasal administration of geraniol complexed to cyclodextrins allowed its selective nose-to-brain delivery [181].

The innovative formulations described in Section 4.1 are summarised in Table 1.

### 4.2. Innovative Phytochemical Formulations Designed to Treat Glioma

Among the most common brain cancers, glioma is characterised by the highest mortality [182]. Gliomas can be treated by surgical resection followed by concomitant chemotherapy; however, unfortunately, this strategy appears unable to efficaciously combat disease progression [183]. Indeed, it is very difficult to completely remove this tumour by resection, because its infiltration and specific growth locations do not allow us to easily distinguish tumour tissue from healthy brain tissue [184]. Moreover, the chemotherapeutic agents can induce serious side effects on healthy cells of the body, showing, on the other hand, poor aptitude for crossing the BBB and penetrating the brain [185].

#### 4.2.1. Curcumin Involved in the Design of New Formulations for Glioma Treatment

In Section 2.2, we reported that the ability of curcumin to recognise and kill the CSCs may be very useful in countering the growth, invasiveness and metastasis of tumours. For this reason, curcumin has been studied as a chemosensitizer, able to increase the therapeutic efficacy of chemotherapeutic agents [186,187]. On the other hand, the ability of curcumin to reach therapeutic amounts in the CNS is compromised by its instability in physiologic fluids, its capacity to be rapidly metabolised in the body, and by its poor solubility, which is related to very low bioavailability [20,188].

**Figure 8:**
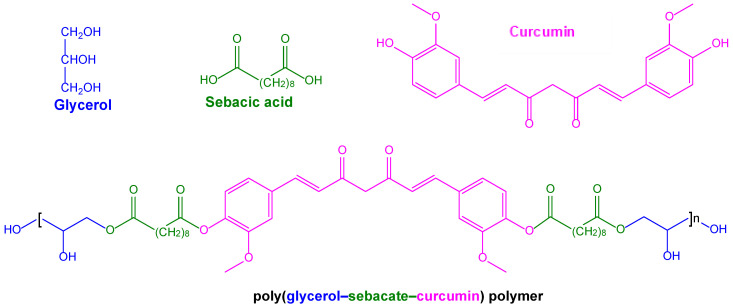
Poly(glycerol–sebacate–curcumin) polymer and the monomers used for its synthesis.

Considering these aspects, several strategies were proposed to increase the potential anticancer activity of curcumin in the CNS, both as a therapeutic agent by itself and as a co-administered chemosensitizer. A first example constitutes the chemical conjugation of curcumin into the biocompatible and biodegradable polymer poly(glycerol–sebacate) to obtain polymers based on poly(glycerol–sebacate–curcumin) unities (Figure 8). In vitro, this system induced a constant long-term release of curcumin, and killed the glioblastoma U-87 and neuroblastoma T-98 cells. Thus, the use of this type of polymer has been proposed for the local treatment of brain tumours [189].

**Figure 9:**
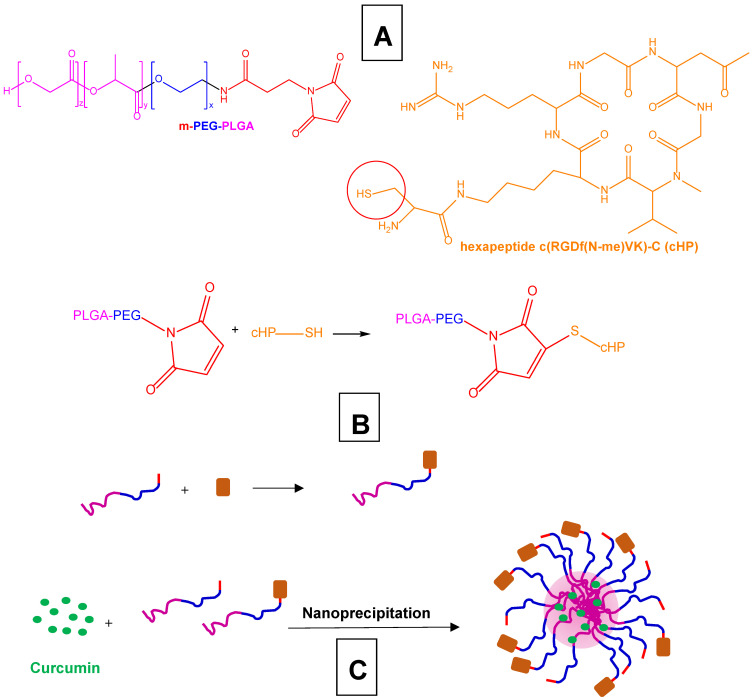
(**A**) Chemical structures of maleimide-poly(ethylene glycol)-poly(lactic-co-glycolide) (m-PEG-PLGA) and cyclic hexapeptide c(RGDf(N-me)VK)-C (cHP). (**B**) These molecules can be easily coupled in order to obtain the cHP-PEG-PLGA structures. (**C**) m-PEG-PLGA and cHP-PEG-PLGA in the presence of curcumin allow to obtain by nanoprecipitation loaded curcumin nanoparticles decorated at their surface with cyclic hexapeptide cHP, able to confer glioma targeting properties to NPs.

Alternatively, innovative nanoparticles have been designed in order to target and induce curcumin delivery in glioma cancer cells. The strategy to target the glioma cells was based on the recognition of overexpressed integrins, such as αvβ3, on the cell surface. Such overexpression seems to contribute to the proliferation, migration and survival of cancer cells [190]. In particular, the targeting ability of the nanoparticles was designed considering a derivative of cilengitide, c(RGDf(N-me)V), a small peptide able to target αvβ3 integrin [191]. To decorate the surface of the PLGA nanoparticles, cilengitide was modified by introducing lysine and cysteine, thereby obtaining the esapeptide c(RGDf(N-me)VK)-C (cHP) and yielding the presence of active sulfhydryl to allow the peptide to couple with a polymer carrier composed of maleimide-poly(ethylene glycol)-poly(lactic-co-glycolide) (m-PEG-PLGA). The uncoupled and coupled polymers were used to formulate, through nanoprecipitation, loaded curcumin nanoparticles decorated with cHP (cHP/Cur-NPs) [192] (Figure 9). The efficacy of this system was evaluated on a C6 rat glioma cell line, cell spheres and glioma tissues, showing that the decorated cHP/Cur-NPs exhibited improved binding, uptake and penetration abilities, in comparison to non-targeting NPs. Moreover, the cHP-decorated nanoparticles appeared able to cross the BBB in vivo, and accumulate in a targeted manner in glioma tumour tissues of rats undergoing C6 cell transplantation [192]. The presence of PEG chains on the surface of nanoparticles was designed to confer them “stealth” properties in vivo, allowing them to increase their circulation time [193].

**Figure 10:**
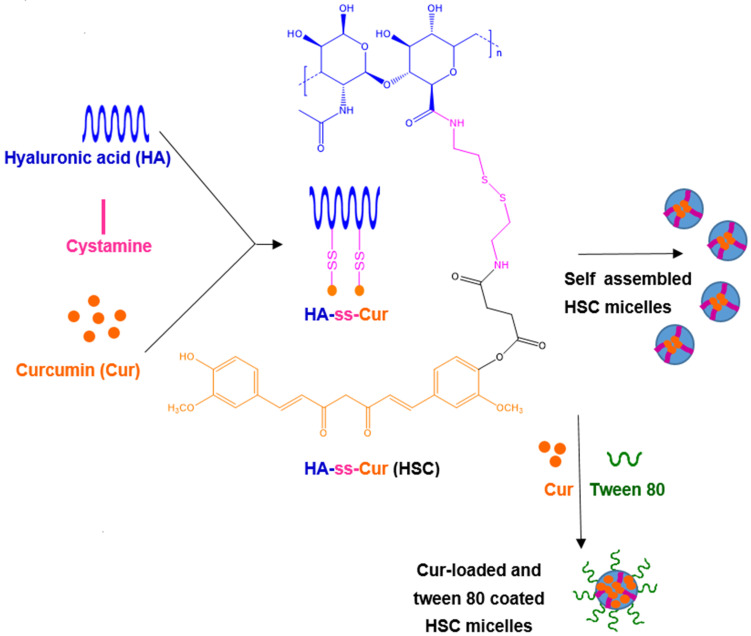
Schematic design of hyaluronic acid–curcumin conjugate via disulphide bond (HSC). In aqueous medium HSC self-assemble as nanomicelles that can be further loaded with curcumin and coated with Tween 80 to induce their ability to cross the BBB. The ability to target and to be internalised by glioma cells is obtained by the presence of hyaluronic acid, a substrate of CD44 markers overexpressed on the surface of cancer cells.

The use of hyaluronic acid as a substrate of the CD44 receptor, which is overexpressed on the surface of gliomas and CSCs (Section 2.2), was proposed as an alternative strategy to achieve the glioma-targeting of nanocarriers conjugated with curcumin [28]. Hyaluronic acid is a polymeric backbone that is biocompatible, biodegradable and non-immunogenic; thus, it is considered suitable for coupling with therapeutic agents or further targeting molecules [28,194]. The ester conjugation of curcumin with the backbone of hyaluronic acid allowed us to obtain amphiphilic conjugates without affecting the pharmacological properties of curcumin [195]. Very recently, a conjugation of hyaluronic acid with curcumin was proposed in the presence of cystamine, via a disulphide bond (Figure 10) [28], which can be broken by glutathione (GSH). In the tumour intracellular microenvironment, GSH appears three orders of magnitude more concentrated than in the extracellular microenvironment. Consequently, the entry of disulphide conjugates into tumour cells can quickly induce the release of conjugated drugs, allowing a prompt and selective bio-responsiveness [196,197]. The hyaluronic acid–curcumin conjugates (HSC) were able to self-assemble as nanomicelles in aqueous environments, with a concomitant increase in the solubility and stability of curcumin. The HSC micelles composed of conjugates of low and medium molecular weight (ranging from 50 to 500 kDa) appeared sensitive to GSH, allowing the curcumin to release, in contrast to the nanomicelles composed of conjugates of high molecular weight (higher than 1000 kDa). Moreover, the low and medium molecular weight HSC conjugates evidenced higher uptake and toxicity towards the G422 glioma cell line, in comparison to the high molecular weight HSC conjugates and plain curcumin [28].

Curcumin was further loaded into the hydrophobic core of HSC micelles. To confer the ability to cross the BBB, the HSC micelles were coated with polysorbate 80 (Tween 80) [198] (Figure 10), which is known to induce the absorption of apolipoprotein E (apoE). Consequently, this favours the transport of nanocarriers into the brain via endocytosis, mediated by the low-density lipoprotein (LDL) receptors expressed on the BBB [199,200]. The HSC micelles loaded with curcumin and coated with Tween 80 evidenced good plasma stability and did not induce haemolysis in erythrocytes; moreover, their uptake and high cytotoxicity towards G422 cells were preserved [28,198]. After intravenous administration to rats, these micelles allowed a plasmatic area under the curve about five folds higher in comparison to the free curcumin to be obtained [198]. Moreover, the Tween 80-coated HSC micelles loaded with Dir, a hydrophobic near-infrared dye, appeared able to effectively accumulate in the brain after intravenous administration to glioma-bearing mice [198].

As reported in Section 2.2, therapies based on curcumin combined with conventional chemotherapeutic agents appear to be promising strategies for cancer eradication, in view of the complementary anti-tumour mechanism of action of these molecules. It is indeed known that curcumin together with conventional anticancer therapies can induce high efficiency in simultaneous inhibition of tumour growth, associated with a reduced treatment-induced toxicity and treatment resistance. The design of appropriate nanocarriers appears of great utility for increasing the bioavailability and bioaccumulation of drugs in a co-delivery strategy with curcumin [201]. Accordingly, a co-delivery design of atorvastatin (ATO) and curcumin was proposed for glioma therapy [194]. This strategy was developed with the formulation of ultra-small nanostructured lipid carriers, bio-conjugated via electrostatic binding with hyaluronic acid, itself conjugated with folic acid or specific peptides (cRGDfK and H7K(R2)2) able to induce targeting and internalization in glioma cells. Folic acid and cRGDfK proteins are the substrates of folate receptors and integrins (αvβ3), respectively, both overexpressed in glioma tumour vasculature and cells; the H7K(R2)2 peptide is characterised by cell penetrating characteristics and behaviour that is responsive to the acidic microenvironment of glioblastoma [202,203,204,205]. Ultra-small nanostructured lipid carriers are constituted by biocompatible and biodegradable materials, resulting in a interaction between solid and liquid lipids, thereby allowing high loading capacities of lipophilic drugs such as ATO and curcumin. The decoration of the surface of these carriers, as above described, appeared appropriate for achieving delivery of the drugs to the targeted glioma cells. Indeed, in vitro, these carriers were able to target and induce cytotoxicity in glioblastoma U-87 cells, showing negligible haemolytic behaviour [194]. After intraperitoneal administration to mice that underwent intracranial injection of U87 glioblastoma, the carriers demonstrated selective targeting to the brain, allowing the prevention of tumour growth and development, in contrast with the treatment using non-encapsulated drugs [194].

A co-delivery curcumin/conventional chemotherapeutic agent strategy against glioma was further proposed by formulating liposomes loaded with temozolomide, curcumin, and doxorubicin. The liposome surface was decorated with the pentapeptide RERMS, then able to allow permeation of the BBB [206]. As expected, an in vitro model of the BBB based on porcine brain-derived capillary endothelial cells suggested the higher ability of the decorated liposomes to cross the BBB, in comparison with non-targeted liposomes. Moreover, the injection of the targeted liposomes (loaded with the three anti-cancer agents) into mice that underwent intracranial injection of human U87 glioblastoma delayed the tumour growth more efficiently than treatment with free compounds [206].

**Figure 11:**
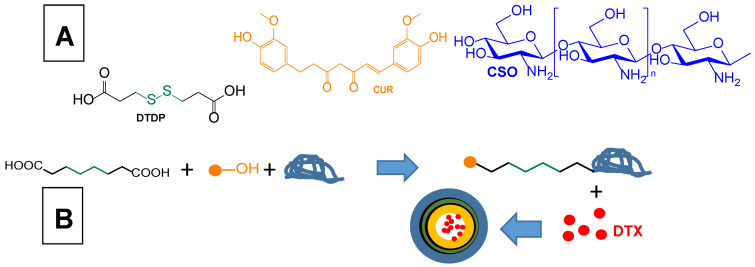
(**A**) Chemical structures of 3,3′-dithiodipropionic acid (DTDP), curcumin (CUR) and chitosan oligosaccharide (CSO). (**B**) The hydrophilic CSO and the hydrophobic drug CUR were coupled by disulphide bonds, via ester conjugation with DTDP, to obtain a polymeric carrier characterised by amphiphilic properties, able to self-assemble as nanomicelles. In particular, docetaxel (DTX) was encapsulated into nanomicelles via self-assembly of the amphiphilic polymer in water.

Again, a co-delivery curcumin/docetaxel (DTX) system was based on curcumin coupling, via a disulphide bond, with chitosan oligosaccharide (CSO) [29]. CSO is obtained as a degradation product of chitosan; it is known to retain the advantages of chitosan, including non-toxicity and biodegradability. Moreover, it is characterised by good BBB penetration ability [207]. As described in Figure 11, the hydrophilic CSO and the hydrophobic drug curcumin were coupled by disulphide bonds, via ester conjugation with 3,3′-dithiodipropionic acid (DTDP), to obtain a polymeric carrier (CSO-ss-curcumin) characterised by amphiphilic properties. CSO-ss-curcumin was able to self-assemble as nanomicelles in aqueous environments, meaning it could be loaded in the presence of docetaxel. Interestingly, curcumin became part of the nanocarrier, constituting the hydrophobic cavity in which DTX is loaded; moreover, it has been demonstrated that in a reductive medium (such as the intracellular environment of glioma), curcumin is rapidly released together with DTX, allowing the two to have their anticancer synergic effects. The efficiency of these nanomicelles as anticancer agents against the glioma C6 cell line was higher than that obtained by the combined free drugs; the intravenous administration of the micelles to C6 tumour-bearing mouse model evidenced their excellent brain-targeting properties in comparison to free drugs [29].

#### 4.2.2. Resveratrol Involved in the Design of New Formulations for Glioma Treatment

Similarly to curcumin, resveratrol has been recognised as an efficient anticancer agent against glioma cells [208,209], showing slight to no harmful side effects in vivo [210]. On the other hand, the therapeutic applications of resveratrol are limited by its poor solubility and inefficient systemic delivery, resulting in very poor bioavailability [211]. Moreover, the presence of the BBB restricts its penetration into the brain and glioma cells, when directly administered in the bloodstream [212].

To increase the BBB penetration of resveratrol, its encapsulation in PEG-PLA nanoparticles (whose surface is decorated with Tf) has been proposed. Tf is indeed known to induce the brain’s uptake via endocytosis [213] by interacting with the TfR, which is selectively expressed on the surface of the brain capillary and overexpressed by glioblastoma cells [214]. The resveratrol-loaded Tf-PEG-PLA nanoparticles evidenced higher uptake and cytotoxicity against the glioma C6 and glioblastoma U-87 cell lines, in comparison to the undecorated resveratrol-loaded PEG-PLA nanoparticles. Moreover, after intraperitoneal administration to glioma-bearing rats, the resveratrol-loaded Tf-PEG-PLA nanoparticles significantly accumulated in brain tumour and decreased its volume, in contrast to free resveratrol [215].

As for other phytochemicals, the co-administration of resveratrol and conventional chemotherapeutics is considered a high-efficacy strategy against glioma. Therefore, a liposome-based approach was very recently proposed for the co-delivery of resveratrol and epirubicin to target glioma cells [30]. Resveratrol was incorporated into the bilayer of liposomes, based on cholesterol and polyethylene glycol distearoylphosphosphatidylethanolamine (PEG-DSPE), whereas epirubicin was loaded in the aqueous compartment. The external liposome PEG chains were coupled via ester and amide conjugation, respectively, to p-aminophenyl-α-D-manno-pyranoside (MAN), to increase BBB penetration, and to germ agglutinin (WGA), in order to target glioma cells [30]. In an in vitro BBB model based on mouse brain microvascular endothelial bEnd.3 cells, the presence of MAN and WGA allow the liposomes to sensibly increase the transport of epirubicin and resveratrol. Moreover, MAN and WGA contributed to enhancing the uptake of these drugs in glioma C6 cell lines, as well as to increasing their ability to induce apoptosis. The same liposomal system showed the highest efficacy in inhibiting the growth of the C6 glioma spheroids. The intravenous administration of MAN-WGA-decorated liposomes to glioma-bearing rats allowed a sensible reduction in tumour growth, and enhanced rat survival [30].

#### 4.2.3. Cinnamaldehyde Involved in the Design of New Formulations for Glioma Treatment

The anticancer synergic effect of cinnamaldehyde and tryptamine has been exploited to create a co-delivery strategy [31]. It is known that cinnamaldehyde can promote tumoral ROS-mediated apoptosis, without inducing toxicity to healthy cells [216]. On the other hand, the chemical instability and water solubility of cinnamaldehyde restrict its potential therapeutic applications [217]. Tryptamine induces specific gliomas’ cytotoxicity via multiple effects [31], and it can be selectively internalised by glioma cells via overexpressed 5-HT receptors on their cell membrane [218].

**Figure 12:**
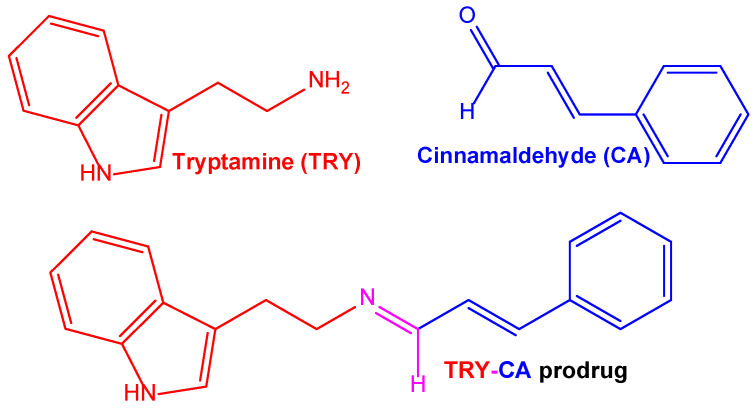
Chemical structures tryptamine, cinnamaldehyde and their prodrug CA-TRY, obtained using conjugation via aldimine condensation.

A system for cinnamaldehyde and tryptamine’s co-delivery was proposed according to the following steps [31]: (i) cinnamaldehyde and tryptamine were conjugated via aldimine condensation, thus obtaining a prodrug (CA-TRY) of both these compounds (Figure 12). The conjugate can indeed release cinnamaldehyde and tryptamine via intracellular endosomal acidolysis of Schiff bases and protonation of tryptamine imidazole rings; and (ii) the hydrophobic CA-TRY prodrug was then auto-assembled as nanospheres using the O/W emulsion solvent evaporation method. The authors hypothesised that the CA-TRY nanospheres can selectively target glioma cells via the overexpressed 5-HT receptors, inducing their intracellular uptake; the following release of cinnamaldehyde and tryptamine allows the drugs to synergically function as anticancer compounds. As the nanoparticles are mainly composed of CA-TRY prodrug, this strategy allowed them to overcome the typical problems related to polymeric characteristics of lipidic nanoparticles, such as low loading, complex formulation and purification methods, and accumulation in the body site of the nanoparticle excipients.

CA-TRY nanoparticles can be selectively internalised in SH-SY5Y neuroblastoma cells, among several tumour cell lines, exhibiting selective apoptotic ability and inducing the growth inhibition of SH-SY5Y multicellular spheroids, which appeared completely destroyed by the synergistic effect of cinnamaldehyde and tryptamine. Moreover, upon intravenous administration to mice, the nanoparticles increased the half-life of the free compounds in the bloodstream and their ability to permeate across the BBB, thus leading to a high compound brain concentration [31].

Table 2 summarises the innovative formulations described Section 4.2.

## 5. Conclusions and Future Directions

Several phytochemicals produced by vegetables for their own protection induce beneficial effects to the human health. These compounds are indeed characterised by a broad spectrum of antioxidant and anti-inflammatory properties exploitable for the prevention and therapy of neurodegenerative diseases and psychotic disorders. Numerous phytochemicals are indeed able to permeate the BBB, appearing to be promising drugs for the treatment of CNS disorders. Moreover, other phytochemical properties contribute to their therapeutic abilities to counteract neurodegeneration. For example, due to their ability to interfere with Aβ accumulation or to induce neuronal protection, curcumin and resveratrol have been proposed as potential agents against AD and PD. Anti-PD properties have also been attributed to geraniol, which is known to promote the survival of dopaminergic neurons by increasing the production of neurotrophic factors. The effects of phytochemicals are not limited to the neuronal disorder, but can also involve interesting therapeutic aspects to counteract tumours. In particular, some phytochemicals appear able to recognise and kill the CSCs involved in chemoresistance, cancer relapse, invasiveness and the development of metastasis. This property was attributed, in particular, to curcumin, but resveratrol and cinnamaldehyde also displayed these features. Accordingly, these compounds appear efficacious against glioma.

Based on their broad beneficial aspects in the brain or against tumours, phytochemicals are proposed as alternative to the current monotherapy approaches, and interesting results are attributed to a co-administration regimen. On the other hand, their poor oral bioavailability and rapid metabolic processes not only reduce their beneficial effects, but also compromise their use for acute or long-term therapies. Several strategies have been proposed to overcome these drawbacks, especially exploiting innovative strategies that are able to promote phytochemicals’ CNS activity. This review summarises these strategies, focusing on those in which phytochemicals, their prodrugs or conjugates are administered or co-administered using nanotechnology-based formulations, themselves supported by conjugation designs. Leptine, wheat germ agglutinin, and TfR represent useful targeting molecules to decorate the surface of phytochemical-loaded nanocarriers to induce permeation of the BBB; alternatively, the Tet-1 peptide can be used to induce uptake in motoneurons, while curcumin can be used to target Aβ deposits. The conjugation of ferulic acid with glycol–chitosan allowed us to obtain a prodrug of these compounds which was able to self-assemble as nanoparticles characterised by prolonged circulation time and neuroprotective effects against spinal cord injury. Geraniol and ursodesoxycholic acid, both neuroactive against PD, conjugated as a prodrug, allowed the encapsulation of solid lipid nanocarriers that upon nasal administration induced prodrug brain-targeting, allowing the co-delivery of the two neuroactive agents to the CNS. Hyaluronic acid has been proposed for the targeting of CD44, which is overexpressed on the surface of gliomas and CSCs. The conjugation of hyaluronic acid with curcumin allowed us to obtain self-assembled nanomicelles with increased stability of curcumin; these were efficaciously taken up in glioma cells. These nanomicelles further loaded with curcumin and coated with Tween 80 (for BBB-crossing ability) displayed effective activity against glioma upon intravenous administration. Again, self-assembled nanomicelles can be obtained via the conjugation of curcumin with chitosan oligosaccharide. In this case, curcumin becomes a part of the nanocarrier, constituting the hydrophobic cavity into which docetaxel can be loaded, in order to achieve co-delivery of the two compounds to glioma cells, resulting in anticancer synergic effects.

The optimization of innovative formulations for the brain-targeting of the phytochemicals can be obtained by means of in vitro models, based on cell lines able to simulate the BBB or the action site of the drugs. The results derived from these in vitro studies can often predict of the in vivo behaviour of these innovative formulations, thus reducing the amount of animal testing required, which is beneficial from an ethical perspective.

Tu summarise, interesting formulations can be obtained through the conjugation of phytochemicals with conventional drugs, allowing us to obtain prodrugs that are able to induce loading increases in nanocarriers designed for nasal administration. These systems appear suitable for ensuring brain-targeting and synergic effects against neurodegenerative diseases. Again, self-assembled nanocarriers can be obtained by producing appropriate conjugations of phytochemicals with biocompatible polymers. The phytochemicals can be used to target the brain, as the part of the self-assembled nanocarriers that can be loaded with conventional drugs, thus allowing us to obtain synergic therapeutic effects to counteract neurodegenerative diseases or glioma. The data reported herein show that these formulations can potentially be designed for either intravenous or oral administration routes.

The formulations described in this review appear potentially efficacious in vivo for the targeting of the brain by agents able to counteract neurodegenerative diseases and tumours; however, poor information is currently available about their effects on the body after long-term treatments. Especially in the latter case, the distribution and accumulation of these nanocarriers at both the central and peripheral level need to be investigated, and the mechanisms of their potential elimination from the body should be elucidated. Knowledge of these aspects may contribute to highlighting interesting perspectives in the battle to treat brain diseases.

## Figures and Tables

**Table 1:** Design of an innovative formulation involving phytochemicals in order to enhance the therapeutic effects against neuronal diseases.

Design	Formulation	In Vitro Cellular or Tissue Models	In Vivo Evaluation	Ref.
Ferulic Acid				
Use of inflammatory cells as carriers of ferulic acid-loaded liposomes decorated with peptide substrates of integrin receptors expressed by cells taken up by the brain in response to neural inflammation	Ferulic acid-loaded liposomes decorated with RGD peptide using conjugation to cholesterol via a succinic spacer	Pro-monocytic U937 cell line for binding and antioxidant studies	Intravenous administration to rat models of brain inflammation (intra-striatal micro-injections of human recombinant IL-1β)	[77,78]
Ferulic acid and glycol chitosan conjugates self-assembled as nanoparticles for a co-delivery strategy of injured spinal cord restoration	Self-assembled nanoparticles obtained using amidic conjugates of ferulic acid with glycol–chitosan	Rat primary neurons for protection studies against glutamate-induced excitotoxicity	Model rats of spinal cord contusion	[26]
Synthesis of prodrugs recognised by L-type amino acid transporters (LAT1)	Prodrug of ferulic acid obtained using amidic or ester conjugation with amino acids recognised by LAT1	Retinal pigment epithelia ARPE-19 cells; mice primary astrocytes from cortex and hippocampi	Intraperitoneal administration to mice	[80,81]
Conjugation of ferulic acid with memantine, which is able to limit the overactivation of N-methyl-D-aspartate receptors	Conjugates of ferulic acid with memantine via a hexamethylene spacer	SH-SY5Y cells as neuronal model	Conjugate proposed for potential use against AD	[82]
Methyl ester derivative of ferulic acid as a prodrug able to increase loading in solid lipid microparticles	Methyl-ferulate-loaded solid lipid microparticles (SLMs) based on tristearin or stearic acid	PC12 cells chosen as a model for neural differentiation	SLMs proposed for potential use as a nasal formulation for methyl-ferulate brain-targeting	[71]
**Caffeic acid**				
Ionic complex between caffeic acid and chitosan carboxymethyl dextran-b-poly(ethylene glycol) for anticancer drug delivery	Nanoparticles of caffeic acid conjugated with chitosan and carboxymethyl dextran-b-poly(ethylene glycol) loading doxorubicin	CT26 cells (murine colorectal carcinoma cell line)		[86]
Caffeic acid conjugated to stearic acid by means of ethylenediamine as a linker to enhance the aqueous dispersibility of micelles	Nanosized-micelles encapsulating budesonide for rectal administration	hTERT-BJ cells (fibroblast cells)	Colitic mouse model	[90]
**Gallic acid**				
Conjugation of gallic acid with gelatine to improve antioxidant properties	Conjugates of gallic acid linked to oxidised dextran to obtain an injectable hydrogel	HT22 cells (neuronal cell line)	Traumatic brain injury mouse model	[98]
**Resveratrol**				
PLGA nanoparticles able to induce the brain’s uptake of resveratrol in cases of PD, due to the presence on the surface of lactoferrin	Resveratrol-loaded PLGA nanoparticles decorated with lactoferrin for intravenous injection	SH-SY5Y cells (neuroblastoma cells)	MPTP-induced PD mice model.	[110]
PLA nanoparticles loaded with resveratrol conjugated with ligands for low-density lipoprotein receptors to cross the BBB via receptor-mediated transcytosis	PLA-coated mesoporous silica nanoparticles	HAPI cells (brain-derived microglial cell line)		[111]
**Curcumin**				
Nanosuspension based on a curcumin prodrug for intramuscular administration with consequent systemic curcumin’s prolonged release	Nanosuspension based on a curcumin prodrug obtained using ester conjugation with decanoic acid		Intramuscular administration to male Wistar rats	[127]
PLGA nanoparticles able to induce curcumin uptake in neurons via the presence of the targeting moiety-Tet-1 peptide on their surface	Curcumin-loaded nanoparticles decorated on their surface with the Tet-1 peptide (HLNILSTLWKYR)	LAG cell lines (mouse fibroblast-like connective tissue) for toxicity studies; GI-1 glioma cells for uptake studies	Nanocarriers are proposed as a potential formulation for the treatment of AD	[125]
Nanoliposomes with high amyloid affinity using surface decoration with curcumin for the AD treatment	SUV obtained in the presence 1,2-dipalmitoyl-sn-glycero-3-phosphothioethanol conjugated to curcumin	HEK cells, control SH-SY5Y cells and hAPP SH-SY5Y cells stably overexpressing the human APP gene causing familial AD	Transgenic mouse AD model (APPxPS1 mice) for injection into the brain of the nanocarrier	[126]
Previous nanoliposomes further implemented with anti-transferrin antibody decoration for BBB targeting	SUV obtained in the presence of phospholipids conjugated with PEG2000 as anchoring points for curcumin or anti-transferrin antibodies	Human brain capillary endothelial cells (hCMEC/D3) as an in vitro model for BBB permeation studies	Proposed as potential formulations for the treatment of AD	[128]
Multifunctional liposomes for co-delivery of curcumin and NGF to the brain	Curcumin-loaded and NGF liposomes obtained in the presence of cardiolipin in their bilayer structure, and decorated with WGA anchored to a PEG spacer	Human brain microvascular endothelial cells (HBMECs) and astrocytes (HAs) for BBB permeation studies; SK-N-MC cells for the Aβ toxicity model	Proposed as potential pharmacotherapy vehicles for AD therapy	[130]
PLGA nanoparticles provided with GSH functionalization to increase the neuronal internalization of curcumin.	Curcumin-loaded PLGA nanoparticles decorated with PEG spacers inserted between polymer and GSH, which was conjugated in the presence of a maleimide moiety	SK-N-SH cells, a human neuroblastoma cell line, as a neuronal internalization model		[131]
PLGA nanoparticles functionalised with the brain-targeting peptide CRT for the co-delivery of curcumin and the Aβ generation inhibitor S1 to the brain	Curcumin and S1-loaded PLGA nanoparticles functionalised with the CRT peptide by conjugation to a PEG spacer	Neuroblastoma SH-SY5Y cells for cytotoxicity studies; brain microvascular bEnd.3 cells as a model for BBB permeation studies	Intravenous administration to male BALB/c nude mice; intraperitoneal injection to AD model (APP/PS1dE9) mice	[132]
PLGA nanoparticles functionalised with B6 peptide for curcumin drug delivery to the CNS	Curcumin-loaded PLGA nanoparticles functionalised with the B6 peptide by conjugation to a PEG spacer	Immortalised mouse hippocampal HT-22 cell line for intracellular studies	Intraperitoneal administration to APP/PS1 transgenic mice for spatial learning and memory capability studies	[133]
Evaluation the uptake of nanoparticles in an in vitro model of Huntington’s disease and cell susceptibility to apoptosis	Nanoparticles based on an amphiphilic conjugate of hyaluronic acid and palmitic acid loaded with curcumin	Striatal-derived immortalised cell line expressing Huntington’s mutation (STHdh^111/111^)		[135]
Effects of curcumin conjugate on spatial memory and oxidative stress in a rat model of multiple sclerosis	Conjugate of curcumin with linoleic acid		Intracerebroventricular injection into male Wistar rats after ethidium bromide treatment as an animal model of multiple sclerosis	[136]
Curcumin encapsulated in bifunctional microemulsions as a targeting agent for Aβ and an inhibitor of fibrils aggregation	Microemulsions based on a modified Pluronic conjugated to the peptide KLVFF for specific binding to Aβ fibrils		Ex vivo permeation study on porcine nasal mucosa; proposed as a potential nasal formulation	[137]
**7,8-dihydroxyflavone**				
Synthesis of a prodrug able to increase the poor oral bioavailability and half-life of dihydroxyflavone for AD treatment	R-13 prodrug	Caco-2 cell line as intestinal model for permeability studies	5XFAD mice as an in vivo model of AD	[138]
Use of R13 prodrug for the oral treatment of peripheral nerve injury	R-13 prodrug		C57B6 wild-type mice	[143]
**Epigallocatechin gallate**				
Formulation of liposomes with the ability to cross the BBB for the co-delivery of EGCG and resveratrol against PD	Resveratrol and EGCG-loaded liposomes decorated on their surface with leptin	Human astrocytes, brain vascular pericytes and brain microvascular endothelial cells for an in vitro model of BBB; SH-SY5Y cells insulted with MPP as an in vitro neurodegenerative model		[147]
**Quercetin**				
Evaluation the ability of α-tocopherol to promote the transport of quercetin across the BBB	Co-administration of quercetin and α-tocopherol		Oral administration to male Sprague Dawley rats	[146]
Water-soluble quercetin conjugate to increase the BBB permeability and to potentially treat cerebral ischemia	Conjugate of quercetin with hyaluronic acid targeted with a penetrating polypeptide, SS31	Human neural cell lines (PC12 and SH-SY5Y cells) and primary cortical neurons	Intraperitoneal injection or through tail vein to male Sprague Dawley rats after middle cerebral artery occlusion as a model of permanent cerebral ischemia	[165]
Quercetin conjugate for improving diabetes-related memory impairment	Conjugate of quercetin with dextran-coated superparamagnetic iron oxide nanoparticles		Oral administration by gavage to male Wistar rats treated with streptozotocin to induce Type 1 diabetes	[166,167]
Quercetin conjugate to enhance the its bioavailability in the brain for the potential treatment of neurodegenerative disorders	Conjugate of quercetin with dextran-coated superparamagnetic iron oxide nanoparticles		Oral administration by gavage to male Wistar rats	[168]
In vitro evaluation of antitoxic properties of free quercetin and quercetin conjugate	Conjugate of quercetin with dextran-coated superparamagnetic iron oxide nanoparticles	Human neural cell lines (PC12 cells)		[169]
In vivo evaluation of anticonvulsant activity of quercetin conjugate	Conjugate of quercetin with superparamagnetic iron oxide nanoparticles coated with β-cyclodextrin and pluronic F68		Intraperitoneal injection to male NMRI mice treated with pentylenetetrazole as a kindling model	[170]
**Genistein**				
Genistein-carrying polypeptide conjugate with propargylamine and Angiopep-2 to enhance the ability to cross the BBB	Genistein-loaded PGA- nanocarrier modified with neuroprotective propargylamine residues together with Angiopep-2	Neuroblastoma SH-SY5Y cell line	Mouse model of AD mice	[178]
**Geraniol**				
Nasal formulation as solid lipid nanoparticles loaded with a prodrug obtained via the conjugation of geraniol with ursodeoxycholic acid	Solid lipid nanoparticles based on compritol loaded with an ester conjugate of geraniol and ursodeoxycholic acid	Rat liver and brain homogenates	Intravenous and nasal administration to rats	[33]
Geraniol encapsulated in chitosan oleate as oral and nasal nanoformulations	Geraniol nanoemulsion obtained in the presence of chitosan oleate as surfactant		Oral and nasal administration to rats	[180]

**Table 2:** Design of innovative formulation involving phytochemicals in order to enhance the therapeutic effects against glioma.

Design	Formulation	In Vitro Cellular Models	In Vivo Evaluation	Ref.
Curcumin				
Conjugation of curcumin into biocompatible and biodegradable polymers for local treatment of tumours	Polymers based on poly(glycerol–sebacate–curcumin) unities	Glioblastoma U-87 and neuroblastoma T-98 cell lines	Proposed for potential use in the local treatment of glioma	[189]
Polymeric nanoparticles decorated with the esapeptide c(RGDf(N-me)VK)-C (cHP) for the targeting of integrins overexpressed by glioma	Curcumin-loaded PEG-PLGA nanoparticles decorated with cHP	C6 rat glioma cell line, C6 cell spheres	Intravenous administration to rats undergoing C6 cell transplantation	[192]
Self-assembled nanoparticles obtained via conjugates of curcumin and hyaluronic acid via a GSH-sensitive disulphide bond. Hyaluronic acid can target glioma cells via CD44 markers. Tween 80 coating induces the BBB permeation.	Curcumin-loaded and Tween 80-coated nanoparticles obtained by self-assembling curcumin–hyaluronic acid conjugates.	Glioblastoma G422 cell line	Intravenous administration to glioma-bearing mice	[28,198]
Co-delivery of curcumin- and atorvastatin-loaded ultra-small nanostructured lipid carriers decorated with folic acid and peptides able to induce targeting and internalization in glioma cells	Curcumin- and atorvastatin-loaded ultra-small nanostructured lipid carriers bio-conjugated via electrostatic binding with hyaluronic acid conjugated with folic acid or specific peptides cRGDfK and H7K(R2)2	Glioblastoma U-87 cells	Intraperitoneal administration to mice that underwent intracranial injection of U87 glioblastoma	[194]
Co-delivery of temozolomide-, curcumin-, and doxorubicin-loaded liposomes decorated with a peptide able to induce the BBB crossing	Temozolomide-, curcumin- and doxorubicin-loaded liposomes decorated with the pentapeptide RERMS	In vitro model of BBB, based on porcine brain-derived capillary endothelial cells	Injection to mice underwenting intracranial injection of human U87 glioblastoma to mice.	[206]
Curcumin coupling via a disulphide bond with chitosan oligosaccharide (CSO) in order to obtain self- assembled nanomicelles loaded with docetaxel for co-delivery to glioma cells	Loaded docetaxel self-assembled nanomicelles based on conjugates of curcumin to CSO by a disulphide bond	Glioma C6 cell line	Intravenous administration to C6 tumour-bearing mouse model	[29]
**Resveratrol**				
Polymeric nanoparticles decorated with transferrin to increase BBB penetration	Resveratrol-loaded PEG-PLGA nanoparticles decorated with transferrin	Glioma C6 and glioblastoma U-87 cell lines	Intraperitoneal administration to glioma-bearing rats	[215]
Co-delivery of resveratrol and epirubicin-loaded polyfunctional liposomes able to cross the BBB and target glioma cells	Resveratrol- and epirubicin-loaded liposomes decorated with p-aminophenyl-α-D-manno-pyranoside (MAN) and germ agglutinin (WGA)	Mouse brain microvascular endothelial (bEnd.3) cells; glioma C6 cell lines; C6 glioma spheroids	Intravenous administration to glioma bearing rats	[30]
**Cinnamaldehyde**				
Co-delivery of cinnamldehyde and tryptamine conjugated to each other via aldimine condensation and self-assembled as nanospheres	Self-assembled nanospheres of a prodrug of cinnamldehyde and tryptamine, obtained using their aldimine condensation	SH-SY5Y neuroblastoma cells; SH-SY5Y multicellular spheroids	Intravenous administration to mice	[31]

## Data Availability

No new data were created or analyzed in this study. Data sharing is not applicable to this article.

## References

[1] Al-Khayri J.M., Sahana G.R., Nagella P., Joseph B.V., Alessa F.M., Al-Mssallem M.Q. (2022). Flavonoids as potential anti-inflammatory molecules: A review. Molecules.

[2] Rinninella E., Cintoni M., Raoul P., Lopetuso L.R., Scaldaferri F., Pulcini G., Miggiano G.A.D., Gasbarrini A., Mele M.C. (2019). Food components and dietary habits: Keys for a healthy gut microbiota composition. Nutrients.

[3] Turini M.E., DuBois R.N. (2002). Primary prevention: Phytoprevention and chemoprevention of colorectal cancer. Hematol. Oncol. Clin. N. Am..

[4] Kerschbaum E., Nüssler V. (2019). Cancer prevention with nutrition and lifestyle. Visc. Med..

[5] Zhang Y.J., Gan R.Y., Li S., Zhou Y., Li A.N., Xu D.P., Li H.B. (2015). Antioxidant phytochemicals for the prevention and treatment of chronic diseases. Molecules.

[6] Van den Brink A.C., Brouwer-Brolsma E.M., Berendsen A.A.M., van de Rest O. (2019). The mediterranean, dietary approaches to stop hypertension (DASH), and mediterranean-DASH intervention for neurodegenerative delay (MIND) diets are associated with less cognitive decline and a lower risk of Alzheimer’s disease-a review. Adv. Nutr..

[7] Leitzmann C. (2016). Characteristics and health benefits of phytochemicals. Forsch. Komplementmed.

[8] Spisni E., Petrocelli G., Imbesi V., Spigarelli R., Azzinnari D., Donati Sarti M., Campieri M., Valerii M.C. (2020). Antioxidant, anti-inflammatory, and microbial-modulating activities of essential oils: Implications in colonic pathophysiology. Int. J. Mol. Sci..

[9] Zhu L., Andersen-Civil A.I.S., Myhill L.J., Thamsborg S.M., Kot W., Krych L., Nielsen D.S., Blanchard A., Williams A.R. (2022). The phytonutrient cinnamaldehyde limits intestinal inflammation and enteric parasite infection. J. Nutr. Biochem..

[10] Feng W., Jin L., Xie Q., Huang L., Jiang Z., Ji Y., Li C., Yang L., Wang D. (2018). Eugenol protects the transplanted heart against ischemia/reperfusion injury in rats by inhibiting the inflammatory response and apoptosis. Exp. Ther. Med..

[11] Rekha K.R., Selvakumar G.P. (2014). Gene expression regulation of Bcl2, Bax and cytochrome-C by geraniol on chronic MPTP / probenecid induced C57BL/6 mice model of Parkinson’s disease. Chem. Biol. Interact..

[12] Rekha K.R., Selvakumar G.P. (2013). Geraniol ameliorates the motor behavior and neurotrophic factors inadequacy in MPTP-induced mice model of Parkinson’ s disease. J. Mol. Neurosci..

[13] Pavan B., Bianchi A., Botti G., Ferraro L., Valerii M.C., Spisni E., Dalpiaz A. (2023). Pharmacokinetic and permeation studies in rat brain of natural compounds led to investigate eugenol as direct activator of dopamine release in PC12 cells. Int. J. Mol. Sci..

[14] Hajinejad M., Ghaddaripouri M., Dabzadeh M., Forouzanfar F., Sahab-Negah S. (2020). Natural cinnamaldehyde and its derivatives ameliorate neuroinflammatory pathways in neurodegenerative diseases. Biomed. Res. Int..

[15] Dalpiaz A., Paganetto G., Botti G., Pavan B. (2020). Cancer stem cells and nanomedicine: New opportunities to combat multidrug resistance?. Drug Discov. Today.

[16] Moselhy J., Srinivasan S., Ankem M.K., Damodaran C. (2015). Natural products that target cancer stem cells. Anticancer Res..

[17] Scarpa E.S., Ninfali P. (2015). Phytochemicals as innovative therapeutic tools against cancer stem cells. Int. J. Mol. Sci..

[18] Xu M.X., Zhao L., Deng C., Yang L., Wang Y., Guo T., Li L., Lin J., Zhang L. (2013). Curcumin suppresses proliferation and induces apoptosis of human hepatocellular carcinoma cells via the wnt signaling pathway. Int. J. Oncol..

[19] Riccardi G., Giosuè A., Calabrese I., Vaccaro O. (2022). Dietary recommendations for prevention of atherosclerosis. Cardiovasc. Res..

[20] Anand P., Kunnumakkara A.B., Newman R.A., Aggarwal B.B. (2007). Bioavailability of curcumin: Problems and promises. Mol. Pharm..

[21] Pavan B., Dalpiaz A., Marani L., Beggiato S., Ferraro L., Canistro D., Paolini M., Vivarelli F., Valerii M.C., Comparone A. (2018). Geraniol pharmacokinetics, bioavailability and its multiple effects on the liver antioxidant and xenobiotic-metabolizing enzymes. Front. Pharmacol..

[22] Thapliyal S., Singh T., Handu S., Bisht M., Kumari P., Arya P., Srivastava P., Gandham R. (2021). A review on potential footprints of ferulic acid for treatment of neurological disorders. Neurochem. Res..

[23] Liu C.S., Chen L., Hu Y.N., Dai J.L., Ma B., Tang Q.F., Tan X.M. (2020). Self-microemulsifying drug delivery system for improved oral delivery and hypnotic efficacy of ferulic acid. Int. J. Nanomed..

[24] Zhang C., Ma W., Zhang Y., Wang Q., Qin C., Du S., Huang L., Ye F., Chen L., Zheng T. (2018). Pharmacokinetics, bioavailability, and tissue distribution study of angoroside c and its metabolite ferulic acid in rat using UPLC-MS/MS. Front. Pharmacol..

[25] Kuo Y.C., Lou Y.I., Rajesh R. (2020). Dual functional liposomes carrying antioxidants against tau hyperphosphorylation and apoptosis of neurons. J. Drug Target..

[26] Wu W., Lee S.Y., Wu X., Tyler J.Y., Wang H., Ouyang Z., Park K., Xu X.M., Cheng J.X. (2014). Neuroprotective ferulic acid (FA)-glycol chitosan (GC) nanoparticles for functional restoration of traumatically injured spinal cord. Biomaterials.

[27] Sozio P., Iannitelli A., Cerasa L.S., Cacciatore I., Cornacchia C., Giorgioni G., Ricciutelli M., Nasuti C., Cantalamessa F., Di Stefano A. (2008). New L-dopa codrugs as potential antiparkinson agents. Arch. Pharm..

[28] Tian C., Asghar S., Xu Y., Chen Z., Zhang M., Huang L., Ye J., Ping Q., Xiao Y. (2018). The effect of the molecular weight of hyaluronic acid on the physicochemical characterization of hyaluronic acid-curcumin conjugates and in vitro evaluation in glioma cells. Colloids Surf. B Biointerfaces.

[29] Liu C., Gao Y., Zhao L., Wang R., Xie F., Zhai G., Liu A. (2022). The development of a redox-sensitive curcumin conjugated chitosan oligosaccharide nanocarrier for the efficient delivery of docetaxel to glioma cells. Ann. Transl. Med..

[30] Kong D., Hong W., Yu M., Li Y., Zheng Y., Ying X. (2022). Multifunctional targeting liposomes of epirubicin plus resveratrol improved therapeutic effect on brain gliomas. Int. J. Nanomed..

[31] Wang Z., Yao J., Guan Z., Wu H., Cheng H., Yan G., Tang R. (2021). pH-triggered small molecule nano-prodrugs emulsified from tryptamine-cinnamaldehyde twin drug for targeted synergistic glioma therapy. Colloids Surf. B Biointerfaces.

[32] Moosavi F., Hosseini R., Saso L., Firuzi O. (2015). Modulation of neurotrophic signaling pathways by polyphenols. Drug Des. Dev. Ther..

[33] de Oliveira Junior E.R., Truzzi E., Ferraro L., Fogagnolo M., Pavan B., Beggiato S., Rustichelli C., Maretti E., Lima E.M., Leo E. (2020). Nasal administration of nanoencapsulated geraniol/ursodeoxycholic acid conjugate: Towards a new approach for the management of Parkinson’s disease. J. Control. Release.

[34] Karvandi M.S., Hesari F.S., Aref A.R., Mahdavi M. (2023). The neuroprotective effects of targeting key factors of neuronal cell death in neurodegenerative diseases: The role of ER stress, oxidative stress, and neuroinflammation. Front. Cell. Neurosci..

[35] Angeloni C., Malaguti M., Prata C., Freschi M., Barbalace M.C., Hrelia S. (2022). Mechanisms underlying neurodegenerative disorders and potential neuroprotective activity of agrifood by-products. Antioxidants.

[36] Das S., Basu S. (2017). Multi-targeting strategies for Alzheimer’s disease therapeutics: Pros and cons. Curr. Top. Med. Chem..

[37] Limanaqi F., Biagioni F., Mastroiacovo F., Polzella M., Lazzeri G., Fornai F. (2020). Merging the multi-target effects of phyto-chemicals in neurodegeneration: From oxidative stress to protein aggregation and inflammation. Antioxidants.

[38] Yadav D.K. (2021). Potential therapeutic strategies of phytochemicals in neurodegenerative disorders. Curr. Top. Med. Chem..

[39] Pogačnik L., Ota A., Ulrih N.P. (2020). An overview of crucial dietary substances and their modes of action for prevention of neurodegenerative diseases. Cells.

[40] Velmurugan B.K., Rathinasamy B., Lohanathan B.P., Thiyagarajan V., Weng C.F. (2018). Neuroprotective role of phytochemi-cals. Molecules.

[41] Moratilla-Rivera I., Sánchez M., Valdés-González J.A., Gómez-Serranillos M.P. (2023). natural products as modulators of Nrf2 signaling pathway in neuroprotection. Int. J. Mol. Sci..

[42] Iside C., Scafuro M., Nebbioso A., Altucci L. (2020). SIRT1 activation by natural phytochemicals: An overview. Front. Pharmacol..

[43] Lee S.H., Lee J.H., Lee H.Y., Min K.J. (2019). Sirtuin signaling in cellular senescence and aging. BMB Rep..

[44] Ege D. (2021). Action mechanisms of curcumin in Alzheimer’s disease and its brain targeted delivery. Materials.

[45] Minocha T., Birla H., Obaid A.A., Rai V., Sushma P., Shivamallu C., Moustafa M., Al-Shehri M., Al-Emam A., Tikhonova M.A. (2022). Flavonoids as promising neuroprotectants and their therapeutic potential against Alzheimer’s disease. Oxid. Med. Cell. Longev..

[46] Behl T., Rana T., Sehgal A., Makeen H.A., Albratty M., Alhazmi H.A., Meraya A.M., Bhatia S., Sachdeva M. (2023). Phyto-chemicals targeting nitric oxide signaling in neurodegenerative diseases. Nitric Oxide.

[47] Fantacuzzi M., Amoroso R., Carradori S., De Filippis B. (2022). Resveratrol-based compounds and neurodegeneration: Recent insight in multitarget therapy. Eur. J. Med. Chem..

[48] Di Giacomo S., Percaccio E., Gullì M., Romano A., Vitalone A., Mazzanti G., Gaetani S., Di Sotto A. (2022). Recent advances in the neuroprotective properties of ferulic acid in Alzheimer’s disease: A narrative review. Nutrients.

[49] Westfall S., Lomis N., Kahouli I., Dia S.Y., Singh S.P., Prakash S. (2017). Microbiome, probiotics and neurodegenerative dis-eases: Deciphering the gut brain axis. Cell. Mol. Life Sci..

[50] Molinero N., Antón-Fernández A., Hernández F., Ávila J., Bartolomé B., Moreno-Arribas M.V. (2023). Gut microbiota, an additional hallmark of human aging and neurodegeneration. Neuroscience.

[51] Saleem A., Qurat-Ul-Ain, Akhtar M.F. (2022). Alternative therapy of psychosis: Potential phytochemicals and drug targets in the management of schizophrenia. Front. Pharmacol..

[52] Kuşoğlu A., Biray Avcı Ç. (2019). Cancer stem cells: A brief review of the current status. Gene.

[53] Niculescu V.F. (2018). The cancer stem cell family: Atavistic origin and tumorigenic development. MOJ Tumor Res..

[54] Dick J.E. (2009). Looking ahead in cancer stem cell research. Nat. Biotechnol..

[55] Peiris-Pagès M., Martinez-Outschoorn U.E., Pestell R.G., Sotgia F., Lisanti M.P. (2016). Cancer stem cell metabolism. Breast Cancer Res..

[56] Kozovska Z., Gabrisova V., Kucerova L. (2014). Colon cancer: Cancer stem cells markers, drug resistance and treatment. Biomed. Pharmacother..

[57] Du L., Wang H., He L., Zhang J., Ni B., Wang X., Jin H., Cahuzac N., Mehrpour M., Lu Y. (2008). CD44 is of functional importance for colorectal cancer stem cells. Clin. Cancer Res..

[58] Szaryńska M., Olejniczak A., Kobiela J., Spychalski P., Kmieć Z. (2017). Therapeutic strategies against cancer stem cells in human colorectal cancer. Oncol. Lett..

[59] Gupta S., Takebe N., Lorusso P. (2010). Targeting the Hedgehog pathway in cancer. Ther. Adv. Med. Oncol..

[60] Yuan X., Wu H., Xu H., Xiong H., Chu Q., Yu S., Wu G.S., Wu K. (2015). Notch signaling: An emerging therapeutic target for cancer treatment. Cancer Lett..

[61] Martin-Orozco E., Sanchez-Fernandez A., Ortiz-Parra I., Ayala-San Nicolas M. (2019). WNT signaling in tumors: The way to evade drugs and immunity. Front. Immunol..

[62] Almanaa T.N., Geusz M.E., Jamasbi R.J. (2012). Effects of curcumin on stem-like cells in human esophageal squamous carcinoma cell lines. BMC Complement. Altern. Med..

[63] Kakarala M., Brenner D.E., Korkaya H., Cheng C., Tazi K., Ginestier C., Liu S., Dontu G., Wicha M.S. (2010). Targeting breast stem cells with the cancer preventive compounds curcumin and piperine. Breast Cancer Res. Treat..

[64] Burgos-Morón E., Calderón-Montaño J.M., Salvador J., Robles A., López-Lázaro M. (2010). The dark side of curcumin. Int. J. Cancer..

[65] Reddivari L., Charepalli V., Radhakrishnan S., Vadde R., Elias R.J., Lambert J.D., Vanamala J.K. (2016). Grape compounds suppress colon cancer stem cells in vitro and in a rodent model of colon carcinogenesis. BMC Complement Altern. Med..

[66] Peng Y., Chu S., Yang Y., Zhang Z., Pang Z., Chen N. (2021). Neuroinflammatory In vitro cell culture models and the potential applications for neurological disorders. Front. Pharmacol..

[67] He W.J., Lv C.H., Chen Z., Shi M., Zeng C.X., Hou D.X., Qin S. (2023). The regulatory effect of phytochemicals on chronic diseases by targeting Nrf2-ARE signaling pathway. Antioxidants.

[68] Bednarek R. (2022). In vitro methods for measuring the permeability of cell monolayers. Methods Protoc..

[69] Toth A.E., Nielsen S.S.E., Tomaka W., Abbott N.J., Nielsen M.S. (2019). The endo-lysosomal system of bEnd.3 and hCMEC/D3 brain endothelial cells. Fluids Barriers CNS.

[70] Sun J., Ou W., Han D., Paganini-Hill A., Fisher M.J., Sumbria R.K. (2022). Comparative studies between the murine immortalized brain endothelial cell line (bEnd.3) and induced pluripotent stem cell-derived human brain endothelial cells for paracellular transport. PLoS ONE.

[71] Botti G., Bianchi A., Pavan B., Tedeschi P., Albanese V., Ferraro L., Spizzo F., Del Bianco L., Dalpiaz A. (2022). Effects of mi-croencapsulated ferulic acid or its prodrug methyl ferulate on neuroinflammation induced by muramyl dipeptide. Int. J. Environ. Res. Public Health.

[72] Kovalevich J., Langford D. (2013). Considerations for the use of SH-SY5Y neuroblastoma cells in neurobiology. Methods Mol. Biol..

[73] Formolo C.A., Williams R., Gordish-Dressman H., MacDonald T.J., Lee N.H., Hathout Y. (2011). Secretome signature of invasive glioblastoma multiforme. J. Proteome Res..

[74] Ghosh S., Basak P., Dutta S., Chowdhury S., Sil P.C. (2017). New insights into the ameliorative effects of ferulic acid in pathophysiological conditions. Food Chem. Toxicol..

[75] Li Y., Liu C., Zhang Y., Mi S., Wang N. (2011). Pharmacokinetics of ferulic acid and potential interactions with Honghua and clopidogrel in rats. J. Ethnopharmacol..

[76] Hassanzadeh P., Arbabi E., Atyabi F., Dinarvand R. (2018). Ferulic acid-loaded nanostructured lipid carriers: A promising nanoformulation against the ischemic neural injuries. Life Sci..

[77] Saini S., Sharma T., Jain A., Kaur H., Katare O.P., Singh B. (2021). Systematically designed chitosan-coated solid lipid nanoparticles of ferulic acid for effective management of Alzheimer’s disease: A preclinical evidence. Colloids Surf. B Biointerfaces.

[78] Qin J., Chen D., Hu H., Cui Q., Qiao M., Chen B. (2007). Surface modification of RGD-liposomes for selective drug delivery to monocytes/neutrophils in brain. Chem. Pharm. Bull..

[79] Qin J., Chen D., Hu H., Qiao M., Zhao X., Chen B. (2007). Body distribution of RGD-mediated liposome in brain-targeting drug delivery. Yakugaku Zasshi.

[80] Puris E., Gynther M., Huttunen J., Auriola S., Huttunen K.M. (2019). L-type amino acid transporter 1 utilizing prodrugs of ferulic acid revealed structural features supporting the design of prodrugs for brain delivery. Eur. J. Pharm. Sci..

[81] Montaser A., Huttunen J., Ibrahim S.A., Huttunen K.M. (2019). Astrocyte-targeted transporter-utilizing derivatives of ferulic acid can have multifunctional effects ameliorating inflammation and oxidative stress in the brain. Oxid. Med. Cell. Longev..

[82] Rosini M., Simoni E., Caporaso R., Basagni F., Catanzaro M., Abu I.F., Fagiani F., Fusco F., Masuzzo S., Albani D. (2019). Merging memantine and ferulic acid to probe connections between NMDA receptors, oxidative stress and amyloid-β peptide in Alzheimer’s disease. Eur. J. Med. Chem..

[83] Pavlíková N. (2023). Caffeic acid and diseases—Mechanisms of action. Int. J. Mol. Sci..

[84] Grabska-Kobylecka I., Kaczmarek-Bak J., Figlus M., Prymont-Przyminska A., Zwolinska A., Sarniak A., Wlodarczyk A., Glabinski A., Nowak D. (2020). The presence of caffeic acid in cerebrospinal fluid: Evidence that dietary polyphenols can cross the blood-brain barrier in humans. Nutrients.

[85] Alam M., Ahmed S., Elasbali A.M., Adnan M., Alam S., Hassan M.I., Pasupuleti V.R. (2022). Therapeutic implications of caffeic acid in cancer and neurological diseases. Front. Oncol..

[86] Lee S.J., Choi K.C., Kang M.S., Oh J.S., Jeong Y., Lee H.C. (2015). Self-organized nanoparticles of caffeic acid conjugated polysaccharide and its anticancer activity. J. Nanosci. Nanotechnol..

[87] Mehtap Kutlu H., Genc L., Guney G. (2013). The impact of caffeic acid loaded solid lipid nanoparticles on cancer treatment. Current Nanoscience.

[88] Šebestík J., Marques S.M., Falé P.L., Santos S., Arduíno D.M., Cardoso S.M., Oliveira C.R., Serralheiro M.L.M., Santos M.A. (2011). Bifunctional phenolic-choline conjugates as anti-oxidants and acetylcholinesterase inhibitors. J. Enzyme Inhib. Med. Chem..

[89] Akomolafe S.F. (2017). The effects of caffeine, caffeic acid, and their combination on acetylcholin-esterase, adenosine deaminase and arginase activities linked with brain function. J. Food Biochem..

[90] Mishra R.K., Ahmad A., Kanika, Kumar A., Vyawahare A., Sakla R., Nadeem A., Siddiqui N., Raza S.S., Khan R. (2023). Caffeic acid-conjugated budesonide-loaded nanomicelle attenuates inflammation in experimental colitis. Mol. Pharm..

[91] Morais L.H., Schreiber H.L., Mazmanian S.K. (2021). The gut microbiota–brain axis in behaviour and brain disorders. Nat. Rev. Microbiol..

[92] Pal S.M., Avneet G., Siddhraj S.S. (2018). Gallic acid: Pharmacogical promising lead molecule: A review. Int. J. Pharmacogn. Phytochem. Res..

[93] Kahkeshani N., Farzaei F., Fotouhi M., Alavi S.S., Bahramsoltani R., Naseri R., Momtaz S., Abbasabadi Z., Rahimi R., Farzaei M.H. (2019). Pharmacological effects of gallic acid in health and diseases: A mechanistic review. Iran. J. Basic Med. Sci..

[94] Gao J., Hu J., Hu D., Yang X. (2019). A role of gallic acid in oxidative damage diseases: A comprehensive review. Nat. Prod. Commun..

[95] Brewer M.S. (2011). Natural antioxidants: Sources, compounds, mechanisms of action, and po-tential applications. Compr. Rev. Food Sci. Food Saf..

[96] Choubey S., Varughese L., Kumar V., Beniwal V. (2015). Medicinal importance of gallic acid and its ester derivatives: A patent review. Pharm. Pat. Anal..

[97] Chen L., Huanga G.L., Lü M.H., Zhang J.X., Xu J., Bai S.P. (2020). Amide derivatives of Gallic acid: Design, synthesis and evaluation of inhibitory activities against in vitro α-synuclein aggregation. Bioorg. Med. Chem..

[98] Zhang D., Chang R., Ren Y., He Y., Guo S., Guan F., Yao M. (2022). Injectable and reactive oxygen species-scavenging gelatin hydrogel promotes neural repair in experimental trau-matic brain injury. Int. J. Biol. Macromol..

[99] Madhusudanan P., Raju G., Shankarappa S. (2020). Hydrogel systems and their role in neural tissue engineering. J. R. Soc. Interface.

[100] Yao M., Gao F., Xu R., Zhang J., Chen Y., Guan F. (2019). A dual-enzymatically crosslinked in-jectable gelatin hydrogel loaded with BMSC improves neurological function recovery of traumatic brain injury in rats. Biomater. Sci..

[101] Bostancieri N., Elbe H., Esrefoglu M., Vardi N. (2022). Cardioprotective potential of melatonin, quercetin and resveratrol in an experimental model of diabetes. Biotech. Histochem..

[102] Bonferoni M.C., Rossi S., Sandri G., Ferrari F. (2017). Nanoparticle formulations to enhance tu-mor targeting of poorly soluble polyphenols with potential anticancer properties. Semin. Cancer Biol..

[103] Fonseca-Santos B., Chorilli M. (2020). The uses of resveratrol for neurological diseases treatment and insights for nanotechnology based-drug delivery systems. Int. J. Pharm..

[104] Andrade S., Ramalho M.J., Pereira M.D.C., Loureiro J.A. (2018). Resveratrol Brain Delivery for Neurological Disorders Prevention and Treatment. Front. Pharmacol..

[105] Komorowska J., Wątroba M., Szukiewicz D. (2020). Review of beneficial effects of resveratrol in neurodegenerative diseases such as Alzheimer’s disease. Adv. Med. Sci..

[106] Zhang L.X., Li C.X., Kakar M.U., Khan M.S., Wu P.F., Amir R.M., Dai D.F., Naveed M., Li Q.Y., Saeed M. (2021). Resveratrol (RV): A pharmacological re-view and call for further research. Biomed. Pharmacother..

[107] Wang H., Jiang T., Li W., Gao N., Zhang T. (2018). Resveratrol attenuates oxidative damage through activating mitophagy in an in vitro model of Alzheimer’s disease. Toxicol. Lett..

[108] Bastianetto S., Zheng W.H., Quirion R. (2000). Neuroprotective abilities of resveratrol and other red wine constituents against nitric oxide-related toxicity in cultured hippocampal neu-rons. Br. J. Pharmacol..

[109] Shimazu R., Anada M., Miyaguchi A., Nomi Y., Matsumoto H. (2021). Evaluation of Blood-Brain Barrier Permeability of Polyphenols, Anthocyanins, and Their Metabolites. J. Agric. Food Chem..

[110] Katila N., Duwa R., Bhurtel S., Khanal S., Maharjan S., Jeong J.H., Lee S., Choi D.Y., Yook S. (2022). Enhancement of blood-brain barrier penetration and the neuroprotective effect of resveratrol. J. Control. Release.

[111] Shen Y., Cao B., Snyder N.R., Woeppel K.M., Eles J.R., Cui X.T. (2018). ROS responsive resvera-trol delivery from LDLR peptide conjugated PLA-coated mesoporous silica nanoparticles across the blood-brain barrier. J. Nanobiotechnology.

[112] Intagliata S., Modica M.N., Santagati L.M., Montenegro L. (2019). Strategies to improve resveratrol systemic and topical bioavailability: An update. Antioxidants.

[113] Chao J., Li H., Cheng K.W., Yu M.S., Chang R.C., Wang M. (2010). Protective effects of pinostil-bene, a resveratrol methylated derivative, against 6- hydroxydopamine-induced neuro-toxicity in SH-SY5Y cells. J. Nutr. Biochem..

[114] Chang J., Rimando A., Pallas M., Camins A., Porquet D., Reeves J., Shukitt-Hale B., Smith M.A., Joseph J.A., Casadesus G. (2012). Low-dose pterostilbene, but not resveratrol, is a potent neuromodulator in aging and Alzheimer’s disease. Neurobiol. Aging.

[115] Poulose S.M., Thangthaeng N., Miller M.G., Shukitt-Hale B. (2015). Effects of pterostilbene and resveratrol on brain and behaviour. Neurochem. Int..

[116] Zhu L., Lu F., Zhang X., Liu S., Mu P. (2022). SIRT1 is involved in the neuroprotection of pterostilbene against amyloid β 25–35-induced cognitive deficits in mice. Front. Pharmacol..

[117] Potdar S., Parmar M.S., Ray S.D., Cavanaugh J.E. (2018). Protective effects of the resveratrol analog piceid in dopaminergic SH-SY5Y cells. Arch. Toxicol..

[118] Peñalver P., Belmonte-Reche E., Adán N., Caro M., Mateos-Martín M.L., Delgado M., González-Rey E., Morales J.C. (2018). Alkylated resveratrol prodrugs and metabolites as poten-tial therapeutics for neurodegenerative diseases. Eur. J. Med. Chem..

[119] Belmonte-Reche E., Peñalver P., Caro-Moreno M., Mateos-Martín M.L., Adán N., Delga-do M., González-Rey E., Morales J.C. (2021). Silyl resveratrol derivatives as potential therapeutic agents for neurodegenerative and neurological diseases. Eur. J. Med. Chem..

[120] Grau L., Soucek R., Pujol M.D. (2023). Resveratrol derivatives: Synthesis and their biological activities. Eur. J. Med. Chem..

[121] Wightman E.L., Reay J.L., Haskell C.F., Williamson G., Dew T.P., Kennedy D.O. (2014). Effects of resveratrol alone or in combination with piperine on cerebral blood flow parameters and cognitive performance in human subjects: A randomised, double-blind, place-bo-controlled, cross-over investigation. Br. J. Nutr..

[122] Haq I.-U., Imran M., Nadeem M., Tufail T., Gondal T.A., Mubarak M.S. (2021). Piperine: A review of its biological effects. Phytother. Res..

[123] Johnson J.J., Nihal M., Siddiqui I.A., Scarlett C.O., Bailey H.H., Mukhtar H., Ahmad N. (2011). Enhancing the bioavailability of resveratrol by combining it with piperine. Mol. Nutr. Food Res..

[124] Belkacemi A., Doggui S., Dao L., Ramassamy C. (2011). Challenges associated with curcumin therapy in Alzheimer disease. Expert. Rev. Mol. Med..

[125] Mathew A., Fukuda T., Nagaoka Y., Hasumura T., Morimoto H., Yoshida Y., Maekawa T., Venugopal K., Kumar D.S. (2012). Cur-cumin loaded-PLGA nanoparticles conjugated with Tet-1 peptide for potential use in Alzheimer’s disease. PLoS ONE.

[126] Lazar A.N., Mourtas S., Youssef I., Parizot C., Dauphin A., Delatour B., Antimisiaris S.G., Duyckaerts C. (2013). Curcu-min-conjugated nanoliposomes with high affinity for Aβ deposits: Possible applications to Alzheimer disease. Nanomedicine.

[127] Wei X.L., Han Y.R., Quan L.H., Liu C.Y., Liao Y.H. (2013). Oily nanosuspension for long-acting intramuscular delivery of curcumin didecanoate prodrug: Preparation, characterization and in vivo evaluation. Eur. J. Pharm. Sci..

[128] Mourtas S., Lazar A.N., Markoutsa E., Duyckaerts C., Antimisiaris S.G. (2014). Multifunctional nanoliposomes with curcumin-lipid derivative and brain targeting functionality with potential applications for Alzheimer disease. Eur. J. Med. Chem..

[129] Frielingsdorf H., Simpson D.R., Thal L.J., Pizzo D.P. (2007). Nerve growth factor promotes survival of new neurons in the adult hip-pocampus. Neurobiol. Dis..

[130] Kuo Y.C., Lin C.C. (2015). Rescuing apoptotic neurons in Alzheimer’s disease using wheat germ agglutinin-conjugated and cardi-olipin-conjugated liposomes with encapsulated nerve growth factor and curcumin. Int. J. Nanomed..

[131] Paka G.D., Ramassamy C. (2017). Optimization of curcumin-loaded PEG-PLGA nanoparticles by GSH functionalization: Investigation of the internalization pathway in neuronal cells. Mol. Pharm..

[132] Huang N., Lu S., Liu X.G., Zhu J., Wang Y.J., Liu R.T. (2017). PLGA nanoparticles modified with a BBB-penetrating peptide co-delivering Aβ generation inhibitor and curcumin attenuate memory deficits and neuropathology in Alzheimer’s disease mice. Oncotarget.

[133] Fan S., Zheng Y., Liu X., Fang W., Chen X., Liao W., Jing X., Lei M., Tao E., Ma Q. (2018). Curcumin-loaded PLGA-PEG nanoparticles conjugated with B6 peptide for potential use in Alzheimer’s disease. Drug Deliv..

[134] Liu Z., Gao X., Kang T., Jiang M., Miao D., Gu G., Hu Q., Song Q., Yao L., Tu Y. (2013). B6 peptide-modified PEG-PLA nanoparticles for enhanced brain delivery of neuroprotective peptide. Bioconjugate Chem..

[135] Pepe G., Calce E., Verdoliva V., Saviano M., Maglione V., Di Pardo A., De Luca S. (2020). Curcumin-loaded nanoparticles based on amphiphilic hyaluronan-conjugate explored as targeting delivery system for neurodegenerative disorders. Int. J. Mol. Sci..

[136] Barzegarzadeh B., Hatami H., Dehghan G., Khajehnasiri N., Khoobi M., Sadeghian R. (2021). Conjugated linoleic acid-curcumin attenuates cognitive deficits and oxidative stress parameters in the ethidium bromide–induced model of demyelination. Neurotox. Res..

[137] Phongpradist R., Thongchai W., Thongkorn K., Lekawanvijit S., Chittasupho C. (2022). Surface modification of curcumin microemul-sions by coupling of KLVFF peptide: A prototype for targeted bifunctional microemulsions. Polymers.

[138] Chen C., Wang Z., Zhang Z., Liu X., Kang S.S., Zhang Y., Ye K. (2018). The prodrug of 7,8-dihydroxyflavone development and therapeutic efficacy for treating Alzheimer’s disease. Proc. Natl. Acad. Sci. USA.

[139] Zuccato C., Cattaneo E. (2009). Brain-derived neurotrophic factor in neurodegenerative diseases. Nat. Rev. Neurol..

[140] Ando S., Kobayashi S., Waki H., Kon K., Fukui F., Tadenuma T., Iwamoto M., Takeda Y., Izumiyama N., Watanabe K. (2002). Animal model of dementia induced by entorhinal synaptic damage and partial restoration of cognitive deficits by BDNF and carnitine. J. Neurosci. Res..

[141] Jang S.W., Liu X., Yepes M., Shepherd K.R., Miller G.W., Liu Y., Wilson W., Xiao G., Blanchi B., Sun Y.E. (2010). A selective TrkB agonist with potent neurotrophic activities by 7,8-dihydroxyflavone. Proc. Natl. Acad. Sci. USA.

[142] Liu X., Qi Q., Xiao G., Li J., Luo H.R., Ye K. (2013). O-methylated metabolite of 7,8-dihydroxyflavone activates TrkB receptor and displays antidepressant activity. Pharmacology.

[143] English A.W., Carrasco D., Hoffman D., Isaacson R., Kang S.S., Khan S., Liu X., Ye K. (2022). Oral Treatments with the TrkB ligand prodrug, R13, promote enhanced axon regeneration following peripheral nerve injury. Front Cell. Neurosci..

[144] Pervin M., Unno K., Takagaki A., Isemura M., Nakamura Y. (2019). Function of green tea catechins in the brain: Epigallocatechin gallate and its metabolites. Int. J. Mol. Sci..

[145] Levites Y., Weinreb O., Maor G., Youdim M.B., Mandel S. (2001). Green tea polyphenol (-)-epigallocatechin-3-gallate prevents N-methyl-4-phenyl-1,2,3,6-tetrahydropyridine-induced dopaminergic neurodegeneration. J. Neurochem..

[146] Ferri P., Angelino D., Gennari L., Benedetti S., Ambrogini P., Del Grande P., Ninfali P. (2015). Enhancement of flavonoid ability to cross the blood-brain barrier of rats by co-administration with α-tocopherol. Food Funct..

[147] Kuo Y.C., Wang I.H., Rajesh R. (2021). Use of leptin-conjugated phosphatidic acid liposomes with resveratrol and epigallocatechin gallate to protect dopaminergic neurons against apoptosis for Parkinson’s disease therapy. Acta Biomater..

[148] Banks W.A. (2001). Leptin transport across the blood-brain barrier: Implications for the cause and treatment of obesity. Curr. Pharm. Des..

[149] Lu J., Park C.S., Lee S.K., Shin D.W., Kang J.H. (2006). Leptin inhibits 1-methyl-4-phenylpyridinium-induced cell death in SH-SY5Y cells. Neurosci. Lett..

[150] Li Y., Zhao J., Hölscher C. (2017). Therapeutic potential of baicalein in Alzheimer’s disease and Parkinson’s disease. CNS Drugs.

[151] Tarragó T., Kichik N., Claasen B., Prades R., Teixidó M., Giralt E. (2008). Baicalin, a Prodrug able to reach the CNS, is a prolyl oligopeptidase inhibitor. Bioorg. Med. Chem..

[152] Zhao W.-Z., Wang H.-T., Huang H.-J., Lo Y.-L., Lin A.M.-Y. (2018). Neuroprotective effects of baicalein on acrolein-induced neurotoxicity in the nigrostriatal dopaminergic system of rat brain. Mol. Neurobiol..

[153] Chen M., Peng L., Gong P., Zheng X., Sun T., Zhang X., Huo J. (2021). Baicalein mediates mi-tochondrial autophagy via mir-30b and the NIX/BNIP3 signaling pathway in Parkinson’s disease. Biochem. Res. Int..

[154] Shi J., Li Y., Zhang Y., Chen J., Gao J., Zhang T., Shang X., Zhang X. (2021). Baicalein amelio-rates Aβ-induced memory deficits and neuronal atrophy via inhibition of PDE2 and PDE4. Front. Pharmacol..

[155] Chen B., Luo M., Liang J., Zhang C., Gao C., Wang J., Wang J., Li Y., Xu D., Liu L. (2018). Surface modification of PGP for a neutrophil–nanoparticle co-vehicle to enhance the anti-depressant effect of baicalein. Acta Pharm. Sin. B.

[156] Jia X., Jia M., Yang Y., Wang D., Zhou F., Zhang W., Huang X., Guo W., Cai D., Chen H. (2019). Synthesis of novel baicalein amino acid derivatives and biological evaluation as neuroprotective agents. Molecules.

[157] Dabeek W.M., Marra M.V. (2019). Dietary quercetin and kaempferol: Bioavailability and potential cardiovascular-related bioactivity in humans. Nutrients.

[158] Batiha G.E.-S., Beshbishy A.M., Ikram M., Mulla Z.S., El-Hack M.E.A., Taha A.E., Algammal A.M., Elewa Y.H.A. (2020). The pharmacological activity, biochemical properties, and pharmacokinetics of the major natural polyphenolic flavonoid: Quercetin. Foods.

[159] Dajas F., Abin-Carriquiry J.A., Arredondo F., Blasina F., Echeverry C., Martínez M., Rivera F., Vaamonde L. (2015). Quercetin in brain diseases: Potential and limits. Neurochem. Int..

[160] Porcu E.P., Cossu M., Rassu G., Giunchedi P., Cerri G., Pourová J., Najmanová I., Migkos T., Pilařová V., No-váková L. (2018). Aqueous injection of quercetin: An approach for confirmation of its direct in vivo cardiovascular effects. Int. J. Pharm..

[161] Manta K., Papakyriakopoulou P., Chountoulesi M., Diamantis D.A., Spaneas D., Vakali V., Naziris N., Chat-ziathanasiadou M.V., Andreadelis I., Moschovou K. (2020). Preparation and bio-physical characterization of quercetin inclusion complexes with β-cyclodextrin derivatives to be formulated as possible nose-to-brain quercetin delivery systems. Mol. Pharm..

[162] Dhawan S., Kapil R., Singh B. (2011). Formulation development and systematic optimization of solid lipid nanoparticles of quercetin for improved brain delivery. J. Pharm. Pharmacol..

[163] Pinheiro R.G.R., Granja A., Loureiro J.A., Pereira M.C., Pinheiro M., Neves A.R., Reis S. (2020). RVG29-functionalized li-pid nanoparticles for quercetin brain delivery and Alzheimer’s disease. Pharm. Res..

[164] Guo L., Huang Z., Huang L., Liang J., Wang P., Zhao L., Shi Y. (2021). Surface-modified engineered exosomes attenuat-ed cerebral ischemia/reperfusion injury by targeting the delivery of quercetin towards impaired neurons. J. Nanobiotech..

[165] Cen J., Zhang R., Zhao T., Zhang X., Zhang C., Cui J., Zhao K., Duan S., Guo Y. (2022). A water-soluble quercetin conjugate with triple targeting exerts neuron-protective effect on cerebral ischemia by mitophagy activation. Adv. Healthc. Mater..

[166] Ebrahimpour S., Esmaeili A., Beheshti S. (2018). Effect of quercetin-conjugated superparamagnetic iron oxide nanoparti-cles on diabetes-induced learning and memory impairment in rats. Int. J. Nanomed..

[167] Ebrahimpour S., Esmaeili A., Dehghanian F., Beheshti S. (2020). Effects of quercetin-conjugated with superparamagnetic iron oxide nanoparticles on learning and memory improvement through targeting microRNAs/NF-ΚB Pathway. Sci. Rep..

[168] Enteshari Najafabadi R., Kazemipour N., Esmaeili A., Beheshti S., Nazifi S. (2018). Using superparamagnetic iron oxide nanoparticles to enhance bioavailability of quercetin in the intact rat brain. BMC Pharmacol. Toxicol..

[169] Yarjanli Z., Ghaedi K., Esmaeili A., Zarrabi A., Rahgozar S. (2019). The antitoxic effects of quercetin and quercetin-conjugated iron oxide nanoparticles (QNPs) against H2O2-induced toxicity in PC12 cells. Int. J. Nanomed..

[170] Hashemian M., Ghasemi-Kasman M., Ghasemi S., Akbari A., Moalem-Banhangi M., Zare L., Ahmadian S.R. (2019). Fabrication and evaluation of novel quercetin-conjugated Fe3O4–β-Cyclodextrin nanoparticles for potential use in epilepsy disorder. Int. J. Nanomed..

[171] Bardestani A., Ebrahimpour S., Esmaeili A., Esmaeili A. (2021). Quercetin attenuates neurotoxicity induced by iron oxide nanoparticles. J. Nanobiotech..

[172] Goh Y.X., Jalil J., Lam K.W., Husain K., Premakumar C.M. (2022). Genistein: A review on its anti-inflammatory properties. Front. Pharmacol..

[173] Chen X., Wu Y., Gu J., Liang P., Shen M., Xi J., Qin J. (2020). Anti-invasive effect and pharmacological mechanism of genistein against colorectal cancer. BioFactors.

[174] Thangavel P., Puga-Olguín A., Rodríguez-Landa J.F., Zepeda R.C. (2019). Genistein as potential therapeutic candidate formenopausal symptoms and other related diseases. Molecules.

[175] Sansai K., Na Takuathung M., Khatsri R., Teekachunhatean S., Hanprasertpong N., Koonrungsesomboon N. (2020). Effects of isoflavone interventions on bone mineral density in postmenopausal women: A systematic review and meta-analysis of randomized controlled trials. Osteoporos. Int..

[176] Sarkaki A., Amani R., Badavi M., Moghaddam A.Z., Aligholi H., Safahani M., Haghighizadeh M.H. (2008). Pre-treatment effect of different doses of soy isoflavones on spatial learning and memory in an ovariectomized animal model of Alzheimer’s disease. Pak. J. Biol. Sci..

[177] Duro-Castano A., Nebot V.J., Niño-Pariente A., Armiñán A., Arroyo-Crespo J.J., Paul A., Feiner-Gracia N., Albertazzi L., Vicent M.J. (2017). Capturing “extraordinary” soft-assembled charge-like polypeptides as a strategy for nanocarrier design. Adv. Mater..

[178] Duro-Castano A., Borrás C., Herranz-Pérez V., Blanco-Gandía M.C., Conejos-Sánchez I., Armiñán A., Mas-Bargues C., Inglés M., Miñarro J., Rodríguez-Arias M. (2021). Targeting Alzheimer’s disease with multimodal polypeptide-based nanoconjugates. Sci. Adv..

[179] Mortiboys H., Furmston R., Bronstad G., Aasly J., Elliott C., Bandmann O. (2015). UDCA exerts beneficial effect on mitochondrial dysfunction in LRRK2(G2019S) carriers and in vivo. Neurology.

[180] Bonferoni M.C., Ferraro L., Pavan B., Beggiato S., Cavalieri E., Giunchedi P., Dalpiaz A. (2019). Uptake in the central nervous system of geraniol oil encapsulated in chitosan oleate following nasal and oral administration. Pharmaceutics.

[181] Truzzi E., Rustichelli C., de Oliveira Junior E.R., Ferraro L., Maretti E., Graziani D., Botti G., Beggiato S., Iannuccelli V., Lima E.M. (2021). Nasal biocompatible powder of Geraniol oil complexed with cyclodextrins for neurodegenerative diseases: Physicochemical characterization and in vivo evidences of nose to brain delivery. J. Control. Release.

[182] Price R.L., Chiocca E.A. (2014). Evolution of malignant glioma treatment: From chemotherapy to vaccines to viruses. Neurosurgery.

[183] Gao H. (2016). Progress and perspectives on targeting nanoparticles for brain drug delivery. Acta Pharm. Sin. B.

[184] Qin L., Wang C.Z., Fan H.J., Zhang C.J., Zhang H.W., Lv M.H., Cui S.D. (2014). A dual-targeting liposome conjugated with transferrin and arginine-glycine-aspartic acid peptide for glioma-targeting therapy. Oncol. Lett..

[185] Pardridge W.M. (2007). Brain drug development and brain drug targeting. Pharm. Res..

[186] Zangui M., Atkin S.L., Majeed M., Sahebkar A. (2019). Current evidence and future perspectives for curcumin and its analogues as promising adjuncts to oxaliplatin: State-of-the-art. Pharmacol. Res..

[187] Liu Z., Huang P., Law S., Tian H., Leung W., Xu C. (2018). Preventive effect of curcumin against chemotherapy-induced side-effects. Front. Pharmacol..

[188] Tønnesen H.H. (2002). Solubility, chemical and photochemical stability of curcumin in surfactant solutions. Studies of curcumin and curcuminoids, XXVIII. Pharmazie.

[189] Sun Z.J., Sun B., Tao R.B., Xie X., Lu X.L., Dong D.L. (2013). A poly(glycerol-sebacate-curcumin) polymer with potential use for brain gliomas. J. Biomed. Mater Res. A.

[190] Desgrosellier J.S., Cheresh D.A. (2010). Integrins in cancer: Biological implications and therapeutic opportunities. Nat. Rev. Cancer..

[191] Mas-Moruno C., Rechenmacher F., Kessler H. (2010). Cilengitide: The first anti-angiogenic small molecule drug candidate design, synthesis and clinical evaluation. Anticancer Agents Med. Chem..

[192] Zhang X., Li X., Hua H., Wang A., Liu W., Li Y., Fu F., Shi Y., Sun K. (2017). Cyclic hexapeptide-conjugated nanoparticles enhance curcumin delivery to glioma tumor cells and tissue. Int. J. Nanomed..

[193] Pavan B., Paganetto G., Rossi D., Dalpiaz A. (2014). Multidrug resistance in cancer or inefficacy of neuroactive agents: Innovative strategies to inhibit or circumvent the active efflux transporters selectively. Drug Discov. Today.

[194] Basso J., Mendes M., Silva J., Sereno J., Cova T., Oliveira R., Fortuna A., Castelo-Branco M., Falcão A., Sousa J. (2020). Peptide-lipid nanoconstructs act site-specifically towards glioblastoma growth impairment. Eur. J. Pharm. Biopharm..

[195] Sharma M., Sahu K., Singh S.P., Jain B. (2018). Wound healing activity of curcumin conjugated to hyaluronic acid: In vitro and in vivo evaluation. Artif. Cells Nanomed. Biotechnol..

[196] Balendiran G.K., Dabur R., Fraser D. (2004). The role of glutathione in cancer. Cell Biochem. Funct..

[197] Schafer F.Q., Buettner G.R. (2001). Redox environment of the cell as viewed through the redox state of the glutathione disulfide/glutathione couple. Free Radic Biol Med..

[198] Tian C., Asghar S., Xu Y., Chen Z., Zhang J., Ping Q., Xiao Y. (2018). Tween 80-modified hyaluronic acid-ss-curcumin micelles for targeting glioma: Synthesis, characterization and their in vitro evaluation. Int. J. Biol. Macromol..

[199] Gastaldi L., Battaglia L., Peira E., Chirio D., Muntoni E., Solazzi I., Gallarate M., Dosio F. (2014). Solid lipid nanoparticles as vehicles of drugs to the brain: Current state of the art. Eur. J. Pharm. Biopharm..

[200] Sun W., Xie C., Wang H., Hu Y. (2004). Specific role of polysorbate 80 coating on the targeting of nanoparticles to the brain. Biomaterials.

[201] Peter K., Kar S.K., Gothalwal R., Gandhi P. (2021). Curcumin in Combination with other adjunct therapies for brain tumor treatment: Existing knowledge and blueprint for future research. Int. J. Mol. Cell. Med..

[202] Saul J.M., Annapragada A., Natarajan J.V., Bellamkonda R.V. (2003). Controlled targeting of liposomal doxorubicin via the folate receptor in vitro. J. Control. Release.

[203] Miura Y., Takenaka T., Toh K., Wu S., Nishihara H., Kano M.R., Ino Y., Nomoto T., Matsumoto Y., Koyama H. (2013). Cyclic RGD-linked polymeric micelles for targeted delivery of platinum anticancer drugs to glioblastoma through the blood-brain tumor barrier. ACS Nano.

[204] Zhao B.X., Zhao Y., Huang Y., Luo L.M., Song P., Wang X., Chen S., Yu K.F., Zhang X., Zhang Q. (2012). The efficiency of tumor-specific pH-responsive peptide-modified polymeric micelles containing paclitaxel. Biomaterials.

[205] Zhao Y., Ren W., Zhong T., Zhang S., Huang D., Guo Y., Yao X., Wang C., Zhang W.Q., Zhang X. (2016). Tumor-specific pH-responsive peptide-modified pH-sensitive liposomes containing doxorubicin for enhancing glioma targeting and anti-tumor activity. J. Control. Release.

[206] Gabay M., Weizman A., Zeineh N., Kahana M., Obeid F., Allon N., Gavish M. (2021). Liposomal carrier conjugated to APP-derived peptide for brain cancer treatment. Cell. Mol. Neurobiol..

[207] Zhu L., Li R., Jiao S., Wei J., Yan Y., Wang Z.A., Li J., Du Y. (2020). Blood-brain barrier permeable chitosan oligosaccharides interfere with β-amyloid aggregation and alleviate β-amyloid protein mediated neurotoxicity and neuroinflammation in a dose- and degree of polymerization-dependent manner. Mar. Drugs.

[208] Jiang H., Shang X., Wu H., Huang G., Wang Y., Al-Holou S., Gautam S.C., Chopp M. (2010). Combination treatment with resveratrol and sulforaphane induces apoptosis in human U251 glioma cells. Neurochem. Res..

[209] Castino R., Pucer A., Veneroni R., Morani F., Peracchio C., Lah T.T., Isidoro C. (2011). Resveratrol reduces the invasive growth and promotes the acquisition of a long-lasting differentiated phenotype in human glioblastoma cells. J. Agric. Food Chem..

[210] Yousef M., Vlachogiannis I.A., Tsiani E. (2017). Effects of resveratrol against lung cancer: In vitro and in vivo studies. Nutrients.

[211] Sanna V., Roggio A.M., Siliani S., Piccinini M., Marceddu S., Mariani A., Sechi M. (2012). Development of novel cationic chitosan-and anionic alginate-coated poly(D,L-lactide-co-glycolide) nanoparticles for controlled release and light protection of resveratrol. Int. J. Nanomed..

[212] Behin A., Hoang-Xuan K., Carpentier A.F., Delattre J.Y. (2003). Primary brain tumours in adults. Lancet.

[213] Huang R.Q., Qu Y.H., Ke W.L., Zhu J.H., Pei Y.Y., Jiang C. (2007). Efficient gene delivery targeted to the brain using a transferrin-conjugated polyethyleneglycol-modified polyamidoamine dendrimer. FASEB J..

[214] Calzolari A., Larocca L.M., Deaglio S., Finisguerra V., Boe A., Raggi C., Ricci-Vitani L., Pierconti F., Malavasi F., De Maria R. (2010). Transferrin receptor 2 is frequently and highly expressed in glioblastomas. Transl. Oncol..

[215] Guo W., Li A., Jia Z., Yuan Y., Dai H., Li H. (2013). Transferrin modified PEG-PLA-resveratrol conjugates: In vitro and in vivo studies for glioma. Eur. J. Pharmacol..

[216] Hong S.H., Ismail I.A., Kang S.M., Han D., Kwon B.M. (2016). Cinnamaldehydes in cancer chemotherapy. Phytother. Res..

[217] Zhao H., Xie Y., Yang Q., Cao Y., Tu H., Cao W., Wang S. (2014). Pharmacokinetic study of cinnamaldehyde in rats by GC-MS after oral and intravenous administration. J. Pharm. Biomed. Anal..

[218] Merzak A., Koochekpour S., Fillion M.P., Fillion G., Pilkington G.J. (1996). Expression of serotonin receptors in human fetal astrocytes and glioma cell lines: A possible role in glioma cell proliferation and migration. Brain Res. Mol. Brain Res..

